# Toxin secretion and trafficking by *Mycobacterium tuberculosis*

**DOI:** 10.1038/s41467-021-26925-1

**Published:** 2021-11-15

**Authors:** David Pajuelo, Uday Tak, Lei Zhang, Olga Danilchanka, Anna D. Tischler, Michael Niederweis

**Affiliations:** 1 Department of Microbiology, University of Alabama at Birmingham, 609 Bevill Biomedical Research Building, 845 19th Street South, Birmingham, AL 35294 USA; 2 Department of Microbiology and Immunology, University of Minnesota Twin Cities, Minneapolis, MN 55455 USA; 3 Present Address: University of Colorado Boulder, Jennie Smoly Caruthers Biotechnology Building B255, 3415 Colorado Avenue, Boulder, CO 80303 USA; 4 Present Address: Merck & Co., Inc., Cambridge, MA 02141 USA

**Keywords:** Cell death, Membrane trafficking, Bacterial toxins, Bacterial secretion

## Abstract

The tuberculosis necrotizing toxin (TNT) is the major cytotoxicity factor of *Mycobacterium tuberculosis* (Mtb) in macrophages. TNT is the C-terminal domain of the outer membrane protein CpnT and gains access to the cytosol to kill macrophages infected with Mtb. However, molecular mechanisms of TNT secretion and trafficking are largely unknown. A comprehensive analysis of the five type VII secretion systems of Mtb revealed that the ESX-4 system is required for export of CpnT and surface accessibility of TNT. Furthermore, the ESX-2 and ESX-4 systems are required for permeabilization of the phagosomal membrane in addition to the ESX-1 system. Thus, these three ESX systems need to act in concert to enable trafficking of TNT into the cytosol of Mtb-infected macrophages. These discoveries establish new molecular roles for the two previously uncharacterized type VII secretion systems ESX-2 and ESX-4 and reveal an intricate link between toxin secretion and phagosomal permeabilization by Mtb.

## Abstract (web summary)

The tuberculosis necrotizing toxin (TNT) is the major cytotoxicity factor of *M. tuberculosis* (Mtb). Mtb possesses five type VII secretion systems (ESX). Pajuelo et al. show that the ESX-4 system is required for TNT secretion and that ESX-2 and ESX-4 systems work in concert with ESX-1 to permeabilize the phagosomal membrane and enable trafficking of TNT into the cytoplasm of macrophages infected with Mtb.

## Introduction

**Fig. 1:**
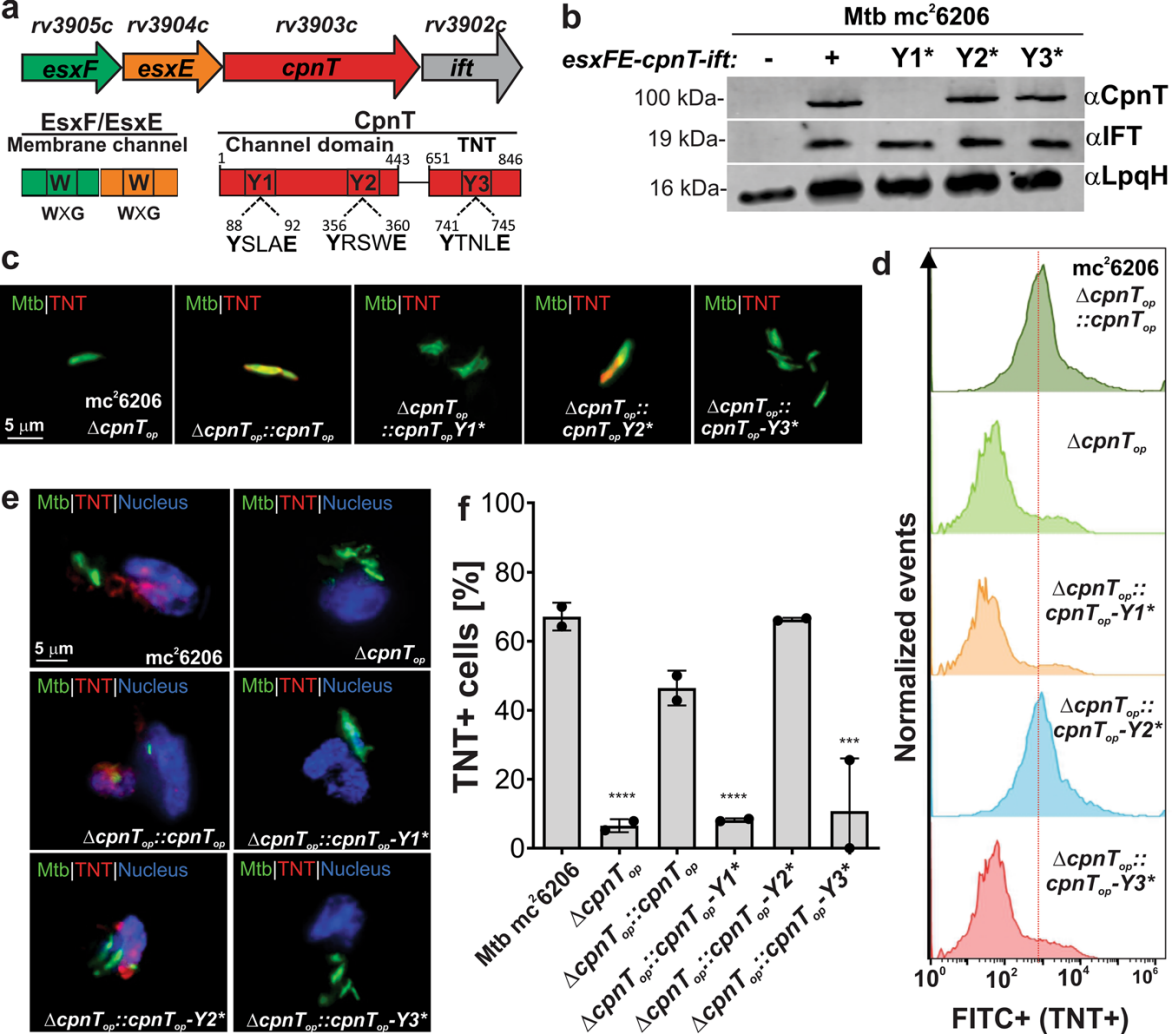
ESX motifs are required for surface accessibility of CpnT in *M. tuberculosis*. **a** The *cpnT* locus of Mtb. The positions of the three putative ESX motifs labeled Y1, Y2, and Y3 of CpnT are indicated. **b** Immunoblot of Mtb whole-cell lysates probed with antibodies for CpnT (anti-TNT antibody) and IFT. LpqH was used as a loading control. **c** Detection of surface-accessible CpnT of the indicated Mtb strains using fluorescence microscopy. The Mtb strains were labeled with the metabolic dye DMN-trehalose (green) and stained with a polyclonal antibody against the TNT domain and Alexa Fluor-594 secondary antibody (red). The yellow color indicates co-localization of TNT with the bacterial cell. **d** Surface accessibility of CpnT by flow cytometry. The indicated Mtb strains were stained with a polyclonal antibody against the TNT domain and Alexa Fluor-488 secondary antibody. The mean fluorescence of Mtb cells is displayed in histograms. **e** Secretion of TNT into the cytosol of Mtb-infected macrophages. The indicated Mtb strains were labeled with the metabolic dye DMN-Trehalose (green) and used to infect THP-1 macrophages at an MOI of 10:1. The macrophages were permeabilized with Triton X-100 48 h after infection, stained with an anti-TNT antibody and Alexa Fluor-594 secondary antibody (red). The macrophage nuclei were stained with DAPI. **f** Quantification of TNT-positive macrophages from images shown in **e**. Macrophages were scored as TNT-positive when distinct red punctae were observed as compared with the Mtb *cpnT* operon deletion mutant (Δ*cpnT*_*op*_). Data are represented as mean ± SEM of at least two independent experiments (*n* ≥ 2). Shown are representative images/blots of at least two independent experiments (*n* ≥ 2). Asterisks indicate significant differences (*p value ≤ 0.05, **p value ≤ 0.01, ***p value ≤ 0.001, ****p value ≤ 0.0001, calculated using the one-way ANOVA with Dunnett’s correction) compared with the Mtb mc^2^6206 strain. Source data are provided in the Source Data file.

Bacterial toxins are central to the pathogenesis and outcome of many infectious diseases^1^. Critical steps of toxin biogenesis are export across the cytoplasmic membrane and, in diderm bacteria, across the outer membrane resulting in cell surface attachment and/or secretion. Toxin export and/or secretion is often mediated by specialized secretion systems. These molecular machines recognize and transport toxins across bacterial and, sometimes, host cell membranes^2–4^. Toxin secretion mechanisms have been described in almost all major bacterial pathogens with the notable exception of *Mycobacterium tuberculosis* (Mtb), the causative agent of tuberculosis. The C-terminal domain of the outer membrane protein CpnT is a novel NAD^+^ glycohydrolase and is the only known exotoxin of Mtb^5^. This toxin gains access to the cytosol of infected cells and induces necroptotic cell death by depleting NAD^+^, and was hence named TNT (tuberculosis necrotizing toxin)^5–7^. However, the molecular mechanisms by which TNT is exported from the bacterial cell and gains access to the cytosol of infected host cells are unknown. We recently showed that the WXG100 proteins EsxE and EsxF form a channel that is essential for CpnT export to the outer membrane^8^. EsxE and EsxF are encoded in the *cpnT* operon (Fig. 1a) and have similarities to the small Esx proteins associated with the five type VII secretion systems of Mtb, indicating that these so-called ESX systems might enable TNT secretion. The ESX-1 system is required to permeabilize the phagosomal membrane^9^, thereby enabling TNT trafficking to the cytosol^6^ and subsequent escape of Mtb from the phagosome^10^ and, eventually, from the dying macrophage. The ESX-3 system is required for siderophore-mediated iron acquisition and for Zn-uptake^11–13^. ESX-5 is present only in slow-growing, pathogenic mycobacteria, is required for uptake of essential nutrients^14^ and exports and/or secretes many PE and PPE proteins, protein families named after motifs containing conserved proline (P) and glutamic acid (E) residues in their N‐terminus^15^. Although the ESX-4 system is required for conjugal transfer of chromosomal DNA in *Mycobacterium smegmatis*^16^ and for growth of *Mycobacterium abscessus* in amoebae^17^, its function in Mtb is unknown. To our knowledge, no molecular functions are known for the ESX-2 system in any mycobacterial species. Thus, it is unknown which ESX system is required for TNT secretion.

Here, we find that both export to the cell surface and secretion of CpnT/TNT into the cytosol of macrophages infected with Mtb require the ESX-4 system. Intriguingly, the ESX-4 system is also involved in the outer membrane localization of the EsxE-EsxF complex. Thus, our study identifies both the inner and outer membrane components of a novel bacterial protein export and secretion machinery. Surprisingly, our comprehensive analysis of the five ESX systems of Mtb also reveals that, in addition to ESX-1, both the ESX-2 and ESX-4 systems are required for rupture of the Mtb-containing phagosome and for access of TNT to the cytosol of macrophages infected with Mtb. Thus, our study identifies not only the system required for the secretion of the only known exotoxin of Mtb, but also establishes new molecular roles for the ESX-2 and ESX-4 systems in phagosomal rupture, a critical step in Mtb pathogenesis.

## Results

### ESX motifs are required for CpnT stability and/or TNT secretion by *M. tuberculosis*

CpnT does not contain a canonical Sec signal peptide in contrast to any other known outer membrane proteins (Supplementary Fig. 1). Since CpnT contains three YXXXE motifs, which were previously shown to be required for secretion of certain ESX-1 substrates^18^, we sought to investigate if they are required for CpnT export by Mtb. These motifs are located at positions Y88, Y356, and Y741 and are referred to hereafter as Y1, Y2, and Y3 motifs, respectively (Fig. 1a, Supplementary Fig. 1). To this end, we used the Mtb ML2016 strain (Supplementary Table 1), which lacks the entire *cpnT* operon, and complemented this strain using integrative expression vectors carrying the *cpnT* operon with either the wt *cpnT* gene or with *cpnT* genes encoding proteins in which the conserved tyrosines of each Y-motif were mutated to alanine (hereafter referred to as Y1*, Y2*, and Y3*). Although expression of the operon producing wt CpnT, CpnT Y2*, and Y3* resulted in similar protein levels, no protein was detected for CpnT Y1* (Fig. 1b). The similar levels of the immunity factor to TNT (IFT) in all strains demonstrated that all operon genes were translated equally (Fig. 1b) and, hence, indicated that the absence of CpnT-Y1* protein resulted from a posttranslational event. Fluorescence microscopy of Mtb strains carrying different *cpnT* alleles using anti-TNT antibodies revealed that the TNT domain is accessible on the cell surface of Mtb (Fig. 1c, Supplementary Fig. 2) as previously shown^6, 8^. As expected, the TNT domain was not detected in Mtb lacking the *cpnT* operon or encoding CpnT with a mutated ESX-Y1* motif, but, surprisingly, TNT was also not detectable on the cell surface in Mtb-encoding CpnT with a mutated ESX-Y3* motif (Fig. 1c, Supplementary Fig. 2), although protein levels and membrane association in subcellular fractionation experiments of CpnT Y3* are similar to that of wt CpnT (Fig. 1b, Supplementary Fig. 3). In order to determine the relative quantities of TNT on the cell surface, we measured the fluorescence of individual Mtb cells by flow cytometry as shown previously^5, 8^. The results of this quantitative analysis are consistent with the fluorescence microscopy experiments: Mtb strains with mutated ESX Y1* and Y3* motifs had no surface-detectable TNT, whereas the Mtb strain with the mutated ESX Y2* motif has wt levels of TNT exposed on the cell surface of Mtb (Fig. 1d).

CpnT protein levels are very low in Mtb grown in vitro, but are strongly elevated in Mtb after infection of macrophages^6^. Hence, we used the human macrophage cell line THP-1 to examine whether the YXXXE motifs affected the secretion of CpnT/TNT in a similar manner as in vitro. Large quantities of TNT in the cytosol of infected THP-1 macrophages were observed when the wt *cpnT* operon or the operon with cpnT-Y2* were expressed, while no secreted TNT was detectable for cpnT-Y1* and cpnT-Y3* (Fig. 1e, f, Supplementary Fig. 4) consistent with the in vitro results. Taken together, these experiments established that mutation of the ESX Y1 and Y3 motifs prevents CpnT from reaching the Mtb cell surface. The absence of CpnT protein with the mutated ESX Y1 motif is likely due to protein degradation, probably caused by protein accumulation and aggregation when protein translocation is impaired^19^. In contrast, protein levels and membrane association of CpnT ESX Y3* are similar to that of wt CpnT, indicating an essential role of the ESX Y3 motif in translocation across the outer membrane. However, for both mutants, we cannot exclude the detrimental effect of the single point mutations Y88A and Y741A on CpnT protein stability.

### The ESX-4 system is essential for surface accessibility of CpnT in *M. tuberculosis*

**Fig. 2:**
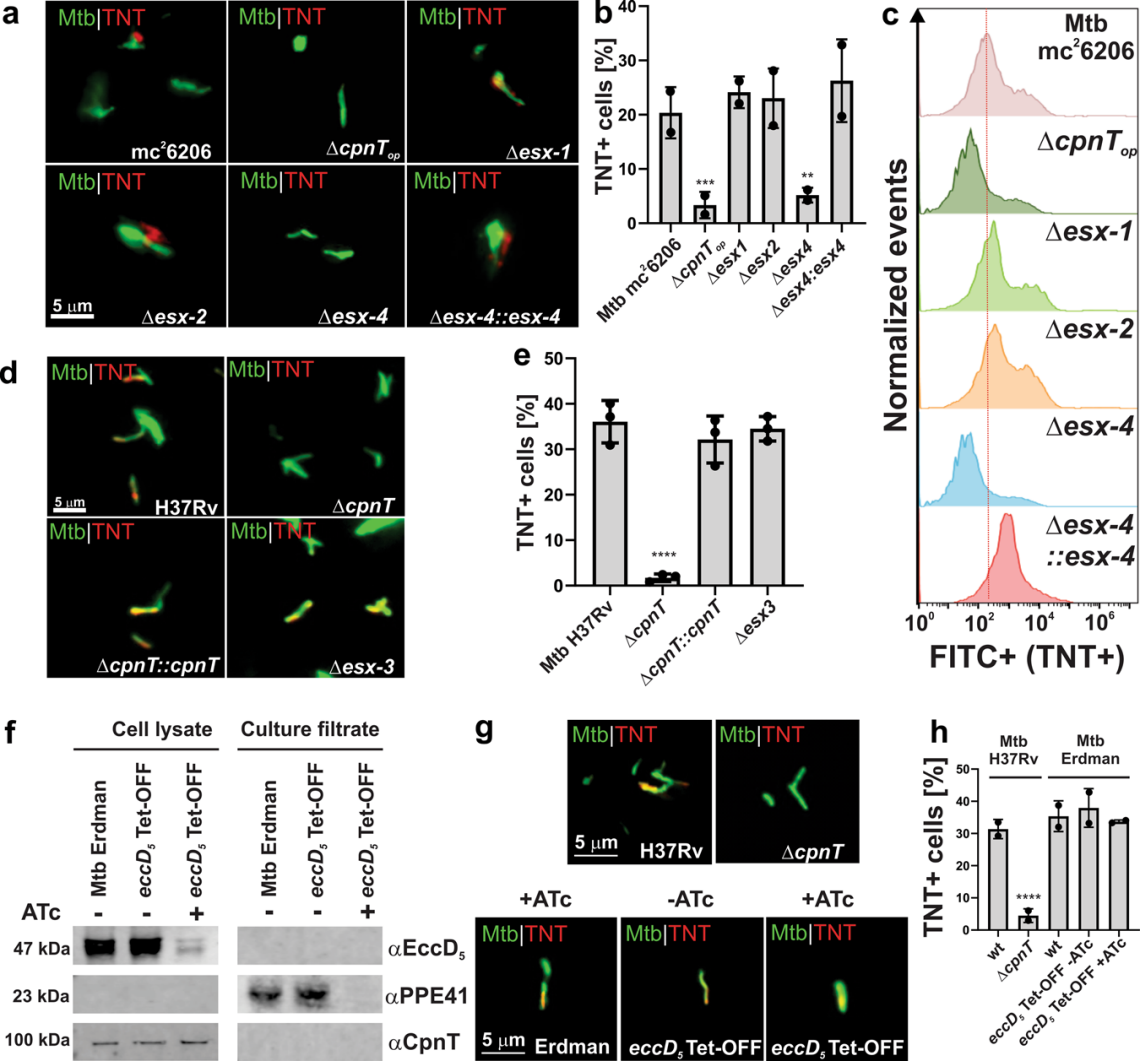
The ESX-4 system is essential for CpnT export and surface accessibility in *M. tuberculosis*. **a** Detection of surface-accessible CpnT in Mtb using fluorescence microscopy. The Mtb strains were labeled with DMN-trehalose (green) and stained with polyclonal anti-TNT and Alexa Fluor-594 secondary antibodies (red). **b** Quantification of TNT-positive Mtb cells from images shown in **a**. Mtb cells were scored as TNT-positive when a red signal was observed as compared with Mtb Δ*cpnT*_*op*_. **c** Surface accessibility of CpnT in Mtb by flow cytometry. The Mtb strains were stained with polyclonal anti-TNT and Alexa Fluor-488 secondary antibodies. The mean fluorescence of Mtb cells is displayed in histograms. **d** Surface-accessibility of CpnT of the Mtb H37Rv *esx-3* deletion mutant and the control strains by fluorescence microscopy. The staining procedure was performed as in **a**. **e** Quantification of TNT-positive Mtb cells from images shown in **d**. Mtb cells were scored as TNT-positive when a red signal was observed as compared to Mtb Δ*cpnT*. **f** Depletion of a functional ESX-5 system in Mtb Erdman. The wt and *eccD*_*5*_ Tet-OFF mutant strains were grown in the presence/absence of anhydrotetracycline hydrochloride (ATc; 100 ng/ml). Proteins from the whole-cell lysates and culture filtrates were analyzed by immunoblotting to detect EccD_5_, PPE41, and CpnT with specific antibodies. **g** Surface-accessibility of CpnT in Mtb Erdman depleted of a functional Esx-5 system using fluorescence microscopy. The staining procedure was performed as in **a**. The wt and Δ*cpnT* strains in the H37Rv background were used as positive and negative controls, respectively. **h** TNT-positive Mtb cells from images shown in **g** were quantified as described in **e**. The Δ*esx-1* strain has a deletion of the RD1 region and lacks the *panCD* genes. Data are represented as mean ± SEM of at least two independent experiments (*n* ≥ 2) and representative images/blots are shown. Asterisks indicate significant differences (*p value ≤ 0.05, **p value ≤ 0.01, *** value ≤ 0.001, ****p value ≤ 0.0001, calculated using the one-way ANOVA with Dunnett’s correction) compared with the corresponding wt strain. Source data are provided in the Source Data file.

The surface accessibility experiments using CpnT with mutations in the putative ESX motifs are consistent with a role of a type VII secretion system in CpnT export but are not conclusive. Therefore, we examined the role of ESX systems in CpnT export and TNT secretion. While YXXXE motifs are required for secretion of ESX-1 and ESX-5 substrates^18^, previous experiments showed that export of CpnT and surface localization of TNT in Mtb does not depend on the ESX-1 secretion system^6^. These results were obtained using the avirulent Mtb mutant mc^2^6206, indicating that the export of CpnT and secretion of TNT are not significantly altered by the deletion of the *leuCD* and *panCD* genes (Supplementary Table 1). This was experimentally confirmed in macrophage infection experiments using the virulent Mtb H37Rv strain, which showed similar TNT secretion (Supplementary Fig. 5) and cytotoxicity (Supplementary Fig. 6) in infected macrophages compared to Mtb mc^2^6206. These results are consistent with previous studies demonstrating that the mechanism of TNT-induced cell death is the same in virulent and avirulent Mtb strains^7, 20^ and with a recent study demonstrating that virulent Mtb H37Rv and the avirulent Mtb Δ*leuD* Δ*panCD* strain have similar replication rates in vitro and in macrophages and elicit similar cytokine response^21^. Hence, we used Mtb mc^2^6206 to obtain mutants with non-functional ESX-2 and ESX-4 systems by constructing unmarked, in-frame deletions of the *eccC2* and *eccC4* genes, respectively (Supplementary Fig. 7a–d). The *eccC* genes encode specific ATPases, which are essential for the function of their respective ESX system^22, 23^. Therefore, deletions in the *eccC2* and *eccC4* genes resulted in the Mtb strains ML2691 and ML2690 (Supplementary Table 1) with non-functional ESX-2 and ESX-4 secretion systems, respectively, from now on referred to as Δ*esx-2* and Δ*esx-4* strains. As the *eccC2* gene is in close proximity to the *esx-1* locus, we examined the secretion of the ESX-1 substrate CFP-10 and showed that the CFP-10 protein levels are identical in the culture filtrates of the *esx-2-* and *esx-4-*deficient strains and its parent strain Mtb mc^2^6206, but no CFP-10 was detected in the Δ*esx-1* mutant (Supplementary Fig. 8). This experiment demonstrated that the eccC2 deletion did not change the activity of the ESX-1 system. In subsequent experiments, the *esx-1* deletion mutant Mtb mc^2^6230 (Supplementary Table 1) was used as a negative control. Fluorescence microscopy revealed that the TNT domain of CpnT is exposed on the cell surface in all Mtb strains except in the strain lacking a functional ESX-4 system and, as expected, in the *cpnT* operon deletion mutant (Fig. 2a, b, Supplementary Fig. 9). TNT surface accessibility was restored when the *esx-4* locus on the cosmid I60 (Supplementary Table 2) was introduced into the Mtb Δ*esx-4* mutant (Figs. 2a, b, Supplementary Fig. 9). Importantly, quantitative analysis of intact Mtb cells by flow cytometry using the TNT antibody as described above confirmed the complete absence of surface accessible CpnT in the Mtb mutant with a non-functional ESX4 system. Expression of the *esx-4* locus on cosmid I60 fully restored surface accessibility of CpnT in Mtb (Fig. 2c). Subcellular fractionation experiments showed a majority of CpnT protein in the membrane fraction in the *esx-4*-deficient Mtb strain (Supplementary Fig. 10). As no surface exposure of the TNT domain was observed in *esx-4*-deficient Mtb, this co-fractionation could result from either a non-functional membrane integration of CpnT or from aggregates of misfolded CpnT due to impaired export as has been observed for other proteins^19^. Taken together, these experiments demonstrate that the ESX-4 secretion system is essential for the translocation of CpnT to the cell surface of Mtb. This result also established CpnT as the first outer membrane protein dependent on a type VII secretion system in Mtb.

### The ESX-3 and ESX-5 systems are not involved in CpnT export in *M. tuberculosis*

To examine the role of the ESX-3 system in CpnT export, we used the *esx-3* deletion mutant Mtb mc^2^7788^13^, a derivative of Mtb H37Rv (Supplementary Table 1). Fluorescence microscopy showed that cell surface exposure of the TNT domain in Mtb H37Rv and the Δ*esx-3* strain is identical, in contrast to that of the *cpnT* deletion mutant (Fig. 2d, e, Supplementary Fig. 11), demonstrating that the ESX-3 system is not involved in CpnT export in Mtb.

To determine whether the export of CpnT is dependent on the ESX-5 system, we used the conditional Mtb Erdman *eccD*_*5*_ TET-OFF mutant, which depletes the essential EccD_5_ transmembrane channel in the presence of anhydrotetracycline (ATc)^24^. The use of this strain is justified because the *cpnT* operon, the *esx-4* locus and all conserved genes of the *esx-5* locus are identical in Mtb H37Rv and Mtb Erdman. We observed 14-fold reduced EccD_5_ levels in the conditional *eccD*_*5*_ mutant grown in the presence of ATc (Fig. 2f), consistent with the previous results^24^. Secretion of the ESX-5 substrate PPE41 into the culture filtrate was completely blocked in the presence of ATc (Fig. 2f), demonstrating that the ESX-5 system is not functional in the conditional *eccD*_*5*_ mutant under repressing conditions and that the low detectable EccD_5_ levels are not sufficient to support the function of the ESX-5 system. The levels of CpnT in the presence of ATc in the conditional *eccD*_*5*_ mutant remained unchanged (Fig. 2f). To examine whether the ESX-5 system plays a role in the functional integration of CpnT in the outer membrane, we assessed TNT exposure on the Mtb cell surface by fluorescence microscopy. As expected, TNT was detectable on the surface of Mtb H37Rv, but not in the Δ*cpnT* mutant, which was used as positive and negative controls, respectively (Fig. 2g, h, Supplementary Fig. 12). Importantly, the addition of ATc did not impair the surface accessibility of CpnT in the *eccD*_*5*_-Tet-OFF mutant (Fig. 2g, h, Supplementary Fig. 12), demonstrating that the ESX-5 secretion system is not involved in the export, outer membrane integration, and surface-exposure of CpnT in Mtb. Taken together with our previous results, these experiments demonstrate that ESX-4 is the sole type VII secretion system required for the export of CpnT and surface exposure of its TNT domain in Mtb.

### The ESX-4 system is involved in EsxF transport to the cell surface of *M. tuberculosis*

**Fig. 3:**
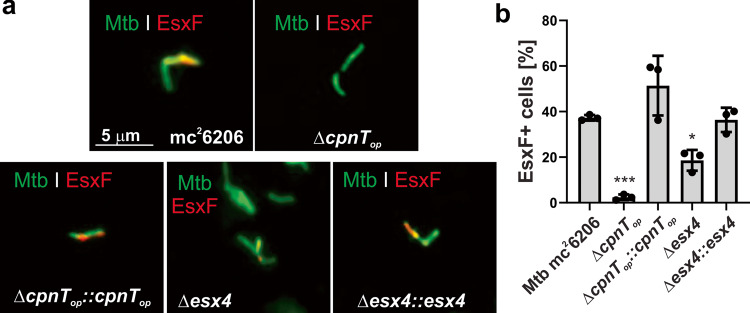
The ESX-4 system is involved in EsxF export in *M. tuberculosis*. **a** Detection of surface-accessible EsxF of the indicated Mtb strains using fluorescence microscopy. The Mtb strains were labeled with the metabolic dye DMN-trehalose (green), and stained with a polyclonal antibody against EsxF and Alexa Fluor-594 secondary antibody (red). The yellow color indicates co-localization of EsxF with the bacterial cell. **b** Quantification of EsxF-positive Mtb cells from images shown in **a**. Mtb cells were scored as EsxF-positive when a red signal was observed as compared with the Mtb *cpnT* operon deletion mutant (Δ*cpnT*_*op*_). Data are represented as mean ± SEM of three independent experiments (*n* = 3) and representative images are shown. Asterisks indicate significant differences (*p value ≤ 0.05, **p value ≤ 0.01, ***p value ≤ 0.001, ****p value ≤ 0.0001, calculated using the one-way ANOVA with Dunnett’s correction) compared with the Mtb mc^2^6206 strain. Source data are provided in the Source Data file.

The small WXG100 proteins EsxE and EsxF are encoded in the *cpnT* operon (Fig. 1a) and form a membrane-spanning pore complex in the outer membrane, which is essential for CpnT export and TNT surface accessibility by Mtb^8^. Considering the requirement of the ESX-4 system for CpnT export in Mtb (Fig. 2), we wondered whether export of the EsxEF complex is also mediated by the ESX-4 system. To test this hypothesis we examined the surface exposure of EsxF in Mtb using fluorescence microscopy and EsxF-specific antibodies^8^. EsxF is clearly detectable on the cell surface in the parent Mtb strain mc^2^6206, but not in the Mtb strain lacking the *cpnT* operon (Fig. 3a, b, Supplementary Fig. 13), consistent with the previous results^8^. Quantitative analysis revealed that the amount of surface-exposed EsxF is reduced by ~50% in an Mtb strain with a non-functional ESX-4 system compared with the parent strain (Fig. 3b), demonstrating an important role of the ESX-4 system in export and surface exposure of the EsxE-EsxF complex. The residual surface-accessible EsxF in the Mtb *esx-4* deletion mutant indicates that alternative pathways for EsxF export and surface translocation exist in addition to ESX-4.

### CpnT trafficking to the cytosol of macrophages infected with *M. tuberculosis* depends on the ESX-1, ESX-2, and ESX-4 systems

**Fig. 4:**
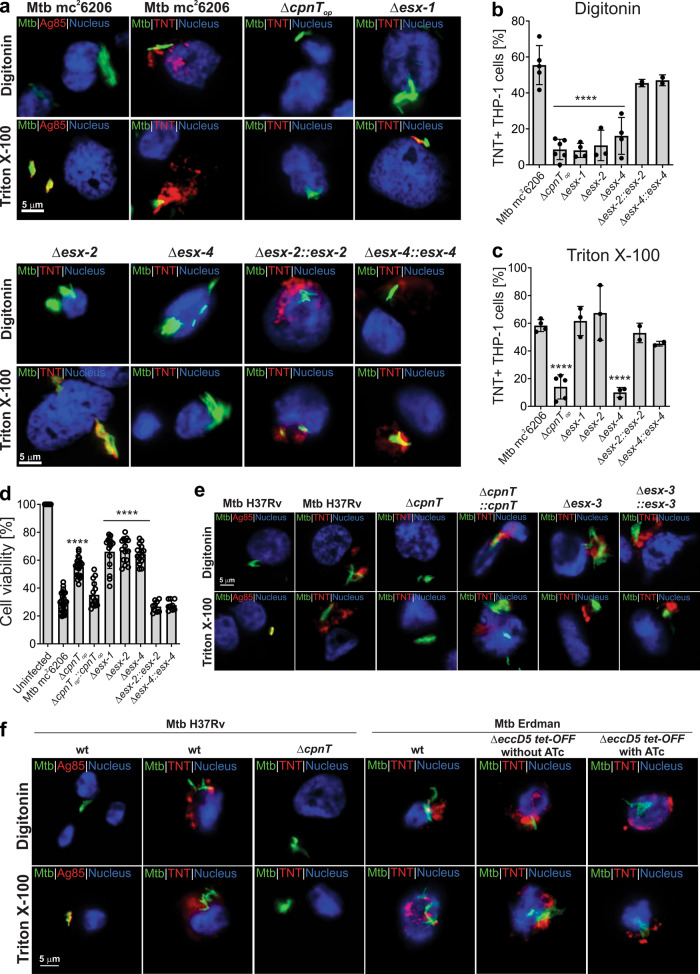
The ESX-1, ESX-2, and ESX-4 systems are required for TNT trafficking into the cytosol of macrophages infected with *M. tuberculosis*. **a** Secretion of TNT into the cytosol of Mtb-infected macrophages. The indicated Mtb strains were labeled with the metabolic dye DMN-Trehalose (green) and used to infect THP-1 macrophages at an MOI of 10:1. After 48 h of infection, the macrophages were permeabilized with digitonin to enable access of antibodies to the cytoplasm, or with Triton X-100 for access to intracellular compartments. Then, cells were stained with an anti-TNT or anti-Ag85 antibody and with an Alexa Fluor-594 secondary antibody (red). The macrophage nuclei were stained with DAPI. **b**, **c** Quantification of TNT-positive macrophages after permeabilization with digitonin (**b**) or Triton X-100 (**c**) from images shown in **a**. Macrophages were scored as TNT-positive when distinct red punctae were observed as compared to the Mtb *cpnT* operon deletion mutant (Δ*cpnT*_*op*_). **d** Cell viability of Mtb-infected macrophages was measured as the total ATP content with a luminescent ATP detection assay kit. **e** Secretion of TNT into the cytosol of infected macrophages in Mtb H37Rv strains. The macrophage infection and staining procedure were performed as described in **a**. **f** Secretion of TNT into the cytosol of infected macrophages in the Mtb H37Rv and Erdman strains. The macrophage infection and staining procedure were performed as described in **a**. When indicated, bacteria were grown in 7H9 + 100 ng/ml anhydrotetracycline (ATc) to pre-deplete EccD5 prior to infection, then ATc was kept in the culture media during the infection. The Δ*esx-1* strain has a deletion of the RD1 region and lacks the *panCD* genes. Data are represented as the mean ± SEM of at least two independent experiments (*n* ≥ 2) and representative images are shown. Asterisks indicate significant differences (*p value ≤ 0.05, **p value ≤ 0.01, ***p value ≤ 0.001, ****p value ≤ 0.0001, calculated using the one-way ANOVA with Dunnett’s correction) compared with the Mtb mc^2^6206 strain. Source data are provided in the Source Data file.

As the ESX-1 system is required for phagosomal permeabilization^9, 25^ and translocation of TNT into the cytosol of macrophages infected with Mtb^6^, we wondered whether other ESX systems might also be involved in this process. To examine this hypothesis we determined the ability of the *esx* mutant Mtb strains to secrete TNT into the cytosol of infected macrophages. To this end, we visualized TNT in THP-1 macrophages infected with the different Mtb strains by fluorescence microscopy after selective permeabilization with the detergents digitonin (plasma membrane only) and Triton X-100 (plasma and phagosomal membranes), as described previously^6, 26^. As a control, we used an antibody specific for the antigen 85 complex (Ag85), which is detected in substantial quantities in the supernatant of Mtb cultures^27^. In the macrophage infection experiments Ag85 was detected only when macrophages were permeabilized with Triton X-100, but not with digitonin (Fig. 4a, Supplementary Fig. 14), demonstrating that Ag85 is contained in the phagosome as we previously described^6^. In contrast, TNT is secreted by Mtb mc^2^6206 into the cytosol of infected THP-1 macrophages consistent with the previous publications^6, 7^. As expected, the *esx-1* mutant produced TNT, but it was confined to the phagosome and did not reach the cytosol of infected macrophages (Fig. 4a–c, Supplementary Fig. 14). This phenotype was observed previously^6^ and is consistent with the known role of ESX-1 in permeabilizing the phagosomal membrane^10, 28, 29^. TNT was undetectable in the cytosol or phagosome of macrophages infected with the *esx-4* deficient strain (Fig. 4a–c, Supplementary Fig. 14). Complementation with the cosmid I60 (Supplementary Table 2) encoding a functional ESX-4 system restored the wt phenotype, confirming the in vitro experiments described above showing that the ESX-4 system is required for CpnT export and surface exposure (Fig. 2). Unexpectedly, we did not detect TNT in the cytosol of macrophages infected with the *esx-2* mutant after permeabilization with digitonin. However, permeabilization with Triton X-100 revealed TNT around the Mtb *esx-2* deficient cells (Fig. 4a–c, Supplementary Fig. 14). This result suggested that TNT was secreted but trapped in the phagosome of the Mtb *esx-2*-deficient strain similar to the Δ*esx-1* strain. Complementation of the *esx-2*-deficient strain with a functional ESX-2 system using the cosmid I106 (Supplementary Table 2) reverted this phenotype and enabled TNT access to the cytosol (Fig. 4a–c, Supplementary Fig. 14). In this regard, it is important to note that the *esx-2* and *esx-4-*deficient Mtb strains retained their ability to produce phthiocerol dimycocerosate (PDIM) (Supplementary Figure 7e), a cell wall glycolipid of Mtb known to be frequently lost during in vitro culture and that has important functions in Mtb pathogenesis, such as in phagosomal membrane damage and rupture, protection from nitric oxide-dependent killing^30, 31^ and outer membrane protein function^32^. The Mtb H37Rv Δ*esx-3* strain and the Mtb conditional *esx-5* mutant under repressing conditions, which impaired the function of the ESX-5 system (Fig. 2f–h), secreted TNT into the cytosol in a similar manner as the parent strain (Fig. 4e, f, Supplementary Figs. 15, 16). These results are consistent with the in vitro experiments and ruled out contributions of the ESX-3 and ESX-5 systems to TNT secretion and trafficking by Mtb. Taken together, these results show that the ESX-1 and ESX-2 systems cannot substitute for each other and that the activities of both ESX systems are required for TNT trafficking into the cytosol of macrophages infected with Mtb.

### The ESX-1, ESX-2, and ESX-4 systems are required for TNT-mediated cytotoxicity in *M. tuberculosis*

The NAD^+^ glycohydrolase TNT is only toxic when it has access to NAD^+^ in the cellular cytosol^6^. Thus, we hypothesized that Mtb ESX mutants with impaired phagosomal permeabilization have reduced cytotoxicity in macrophages owing to impaired toxin trafficking. To test this hypothesis, we measured the viability of THP-1 macrophages infected with Mtb mc^2^6206 and the *esx-1*, *esx-2,* and *esx-4*-deficient mutants. The Mtb mc^2^6206 strain has the same cytotoxicity as its virulent Mtb H37Rv parent strain (Supplementary Figs. 5, 6), consistent with a previous report^21^. The cytotoxicity of the Mtb strain lacking the *cpnT* operon in macrophages was largely reduced (Fig. 4d). Complementation with the *cpnT* operon completely restored its cytotoxicity to wt levels (Fig. 4d), in accordance with previous results demonstrating that CpnT and, specifically the catalytic activity of its TNT domain, is the major cytotoxicity factor in macrophages infected with Mtb^6, 7^. Importantly, the cytotoxicity of the Δ*esx-1*, Δ*esx-2,* and Δ*esx-4* strains was reduced to a similar level as that of the Δ*cpnT* strain (Fig. 4d). Cytotoxicity was restored when the respective Mtb mutants were complemented with cosmids I106 and I60 (Supplementary Table 2) containing the *esx-2* or *esx-4* loci, respectively (Fig. 4d). These results are consistent with the previous experiments that the ESX-1, -2, and -4 systems are required for CpnT/TNT to reach the cytosol of macrophages infected with Mtb.

### The ESX-1, ESX-2, and ESX-4 systems are required for phagosomal permeabilization in *M. tuberculosis*

**Fig. 5:**
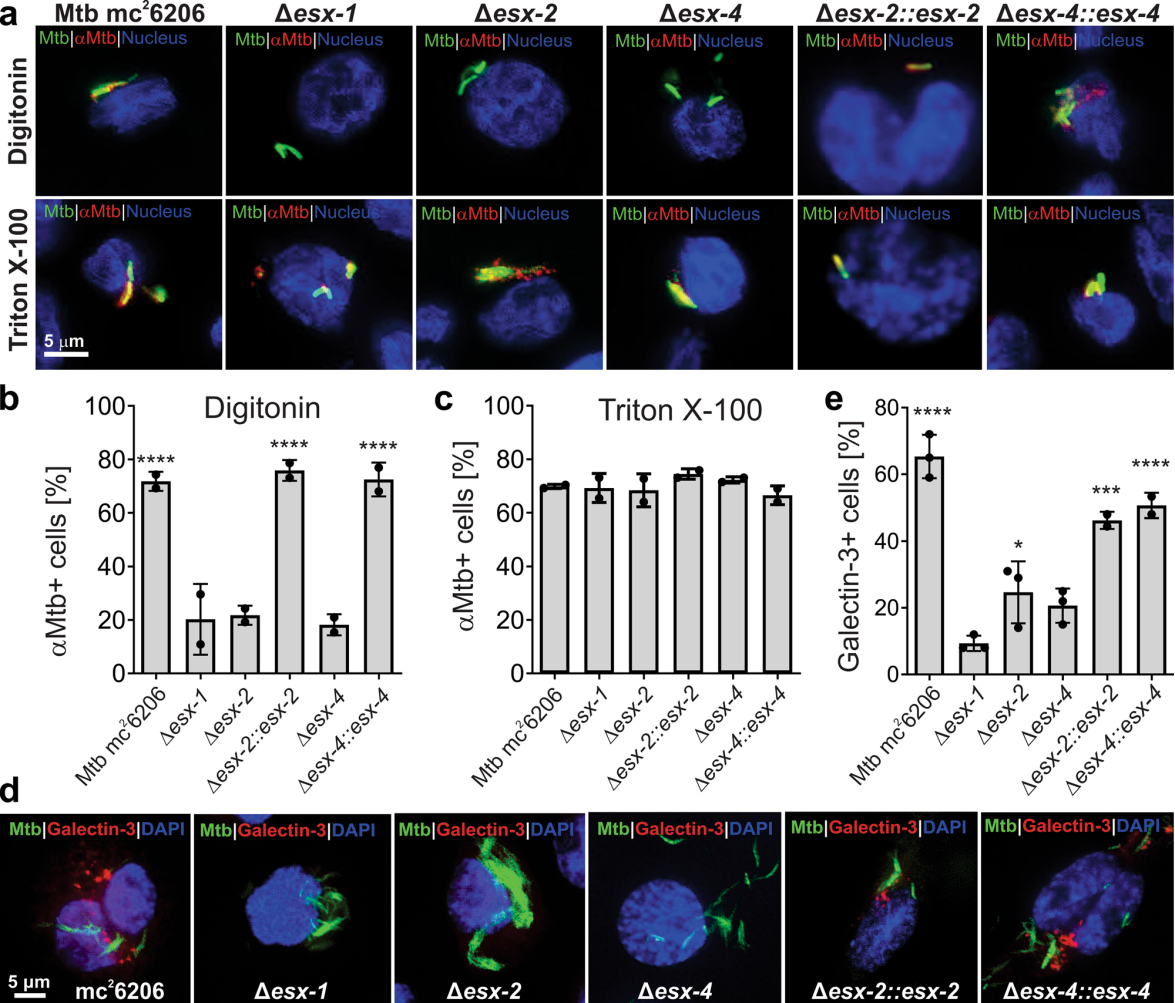
The ESX-1, ESX-2, and ESX-4 systems are required for phagosomal rupture by *M. tuberculosis*. **a** Detection of phagosomal rupture in infected macrophages by using Mtb-specific antibodies. The indicated Mtb strains were labeled with the metabolic dye DMN-Trehalose (green) and used to infect THP-1 macrophages at an MOI of 10:1. After 48 h of infection, the macrophages were permeabilized with digitonin to enable access of antibodies to the cytoplasm, or with Triton X-100 for access to intracellular compartments. Then, cells were stained with an anti-Mtb antibody (αMtb) and with an Alexa Fluor-594 secondary antibody (red). The macrophage nuclei were stained with DAPI. **b**, **c** Quantification of αMtb-positive macrophages after permeabilization with digitonin (**b**) and Triton X-100 (**c**) from images shown in **a**. Macrophages were scored as αMtb-positive when distinct red punctae were observed as compared to the Mtb *esx-1* mutant treated with digitonin. **d** Detection of ruptured phagosomes in macrophages infected with Mtb. The indicated Mtb strains were labeled with the metabolic dye DMN-Trehalose (green) and used to infect THP-1 macrophages at an MOI of 10:1. After 48 h of infection, the macrophages were permeabilized with Triton X-100 and stained with an antibody against the phagosome rupture marker Galectin-3 and with an Alexa Fluor-594 secondary antibody (red). **e**. Quantification of Galectin-3-positive macrophages from images shown in **d**. Macrophages were scored as Galectin-3-positive when distinct red punctae were observed as compared to the Mtb *esx-1* mutant. The Δ*esx-1* strain has a deletion of the RD1 region and lacks the *panCD* genes. Data are represented as mean ± SEM of at least two independent experiments (*n* ≥ 2) and representative images are shown. Asterisks indicate significant differences (*p value ≤ 0.05, **p value ≤ 0.01, ***p value ≤ 0.001, ****p value ≤ 0.0001, calculated using the one-way ANOVA with Dunnett’s correction) compared with the Mtb Δ*esx-1* strain. Source data are provided in the Source Data file.

Based on the observation that TNT access to the host cytosol requires the ESX-2 system, we hypothesized that the ESX-2 system might have a similar function as ESX-1 in rupturing the phagosome^9, 10^. To exclude any CpnT/TNT-specific effect, we examined the role of the Mtb ESX systems in cytosolic protein trafficking using an antiserum against Mtb proteins in the tuberculin purified protein derivative (PPD)^33^. To this end, the membranes of infected macrophages were selectively permeabilized using digitonin as described above. Antibodies in the PPD-antiserum detected proteins after treatment with digitonin, indicating that the phagosomal membrane is permeabilized by the parent strain Mtb mc^2^6206. This is consistent with the previously observed cytoplasmic access of TNT in Mtb. Interestingly, the detected proteins appear to be in close proximity with Mtb cells in contrast to TNT, which spreads throughout the cytoplasm of infected macrophages (Fig. 5a–c, Supplementary Fig. 17). As expected, no Mtb proteins were detected after digitonin treatment of the macrophages infected with the Mtb strain lacking the ESX-1 system (Fig. 5a–c, Supplementary Fig. 17). This result is consistent with many reports that Mtb requires the ESX-1 system to rupture the phagosome and our previous results that ESX-1 is necessary for TNT to access the cytosol of infected macrophages. This experiment also confirms that phagosomal permeabilization can be determined by access of antibodies to cell-associated or secreted proteins of Mtb in addition to detecting TNT as shown in Fig. 4a. Strikingly, no Mtb proteins were detected in digitonin-permeabilized macrophages infected with the *esx-2-* and *esx-4-*deficient strains, whereas both strains produced detectable proteins following Triton X-100 permeabilization (Fig. 5a–c, Supplementary Fig. 17). Access of antibodies to Mtb proteins in macrophages treated with digitonin was restored by expressing the *esx-2* and *esx-4* loci in the respective Mtb mutants using the cosmids I106 and I60 (Supplementary Table 2), respectively (Fig. 5a, b, Supplementary Fig. 17). As the protein levels of the ESX-1 substrate CFP-10 are identical in the culture filtrates of the *esx-2-* and *esx-4-*deficient strains and its parent strain Mtb mc^2^6206 (Supplementary Fig. 8), we concluded that the lack of phagosomal permeabilization in macrophages infected with the *esx-2-* and *esx-4-*deficient strains are not due to a compromised ESX-1 system. Hence, these results demonstrate that both the ESX-2 and ESX-4 systems are required, in addition to ESX-1, for permeabilization of the phagosomal membrane and protein trafficking between the phagosome and the cytosol of macrophages infected with Mtb. These results reveal the first known molecular function of the ESX-2 system in Mtb, and show that the ESX-4 system has at least two functions: export and secretion of CpnT/TNT by Mtb and permeabilization of the phagosome together with ESX-1 and ESX-2, thus enabling toxin diffusion to the macrophage cytosol.

We suspected that the ESX-2 and ESX-4 systems contribute to rupturing of the phagosomal membrane by membranolytic activity in a similar manner as ESX-1^34, 35^. To test this hypothesis we used galectin-3, a host protein that recognizes lumenal glycans that become exposed to the cytosol when a phagosome is extensive damaged^36–38^. Indeed, macrophages infected with Mtb showed significant membrane damage close to the bacteria as visualized using the galectin-3 antibody (Fig. 5d, e, Supplementary Fig. 18). As expected, galectin-3 staining was absent in macrophages infected with the Δ*esx-1* strain and strongly reduced in macrophages infected with the Δ*esx-2* and Δ*esx-4* strains (Fig. 5d, e, Supplementary Fig. 18). Importantly, galectin-3 staining was restored to wt levels by expressing the *esx-2* and *esx-4* loci using the cosmids I106 and I60 (Supplementary Table 2) in the respective Mtb mutants (Fig. 5d, e, Supplementary Fig. 18), demonstrating that the ESX-2 and ESX-4 systems indeed damage the Mtb-containing phagosome. Quantification of galectin staining revealed that more membrane damage is caused by the ESX-1 system compared to the ESX-2 system (Fig. 5e). In contrast, the permeabilization experiments using TNT and Mtb antibodies showed identical phenotypes for the *esx-1*, *esx-2,* and *esx-4*-deficient strains. Hence, we conclude that the coordinated activity of all three ESX systems is required for phagosomal permeabilization. Collectively, these results demonstrate that the ESX-4 system is not only required for TNT secretion by Mtb, but also has an essential role in permeabilizing the phagosomal membrane, together with ESX-1 and ESX-2, to enable trafficking of TNT and other proteins to the cytosol of macrophages infected with Mtb.

## Discussion

### The type VII secretion system ESX-4 is essential for CpnT export in *M. tuberculosis*

Our study established CpnT as the first bacterial outer membrane protein that is dependent on a type VII secretion system. This is consistent with previous results showing that the small, *cpnT* operon-encoded EsxE-EsxF proteins mediate TNT secretion^8^. Here, we show by fluorescence microscopy and by flow cytometry that the surface accessibility of CpnT in Mtb cells, and hence its translocation across the inner membrane and/or its assembly in the outer membrane, completely depend on the ESX-4 system, whereas inactivation of the other four ESX systems of Mtb showed no phenotype (Fig. 2). The requirement of ESX-4 for CpnT export and TNT surface localization in Mtb was also observed in macrophages (Fig. 4). Our results in Mtb contrast the recent claim that CpnT is a substrate of the ESX-5 system in *Mycobacterium marinum*^39^. Differences in the mechanism of CpnT export might be based on divergent functions of ESX systems in different mycobacteria as previously observed for ESX-1 and ESX-4, which are involved in DNA conjugation in *Mycobacterium smegmatis*^16^, but not in Mtb^40^. It is also possible that *cpnT* overexpression artifacts caused the disparate results in *M. marinum*. In fact, overexpressing the *cpnT* operon in Mtb results in significant amounts of water-soluble CpnT (Supplementary Fig. 3). Importantly, CpnT is fully localized in the membrane fraction of Mtb, as expected for an integral membrane protein, when *cpnT* is expressed from its native chromosomal operon (Supplementary Fig. 10), in accordance with the previous results^5^. Interestingly, interfering with CpnT export either by inactivation of the ESX-4 system or by overwhelming ESX-4 secretion capacity upon overproducing CpnT resulted in the appearance of partially water-soluble CpnT protein. This phenotype may be caused by the accumulation of export-competent precursors as observed for other proteins in the periplasm when their translocation was impaired^19^. Taken together, our study unequivocally identifies the ESX-4 system as the type VII secretion system mediating CpnT export and surface accessibility, and defines the first molecular function for this secretion system in Mtb.

### The molecular functions of the ESX-4—EsxE-EsxF system in CpnT export resemble those of the Sec system and β-barrel assembly machinery in Gram-negative bacteria

**Fig. 6:**
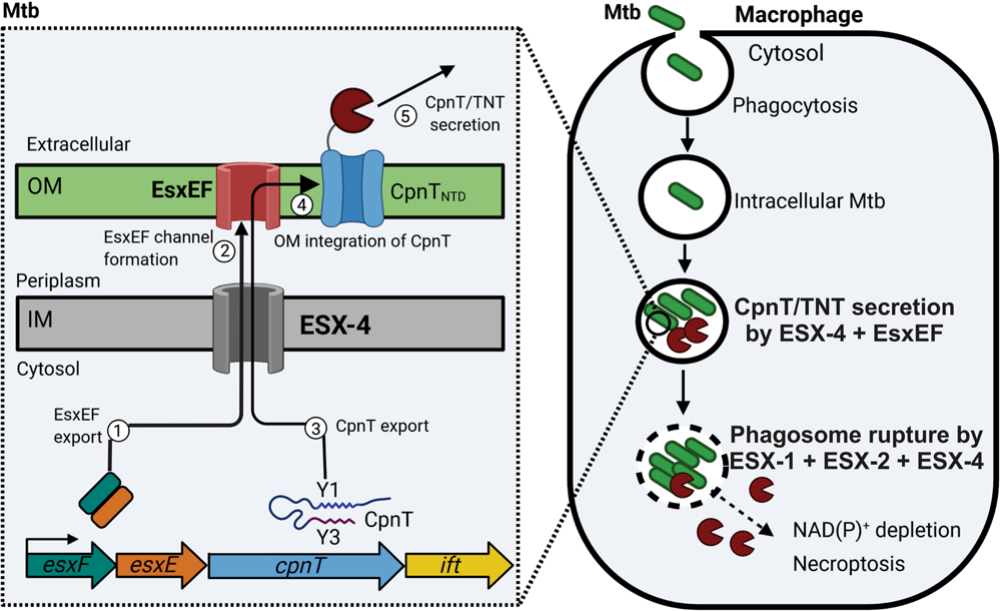
Model of CpnT export and TNT secretion and trafficking by *M. tuberculosis*. After phagocytosis Mtb is trapped inside the phagosome and expresses the *esx-4* genes and the *cpnT* operon. The ESX-4 core complex is assembled in the inner membrane (IM). Then, the EsxEF complex is exported (1) by the ESX-4 system and forms a pore in the outer membrane (OM) (2) as shown previously^8^. CpnT is exported through the ESX-4 system, possibly involving the ESX Y3 motif (3). CpnT is then integrated into the outer membrane by an unknown mechanism (4). After translocation of its C-terminal NAD^+^ glycohydrolase domain to the cell surface, possibly via the ESX motif Y3, CpnT/TNT is secreted (5). CpnT/TNT trafficking into the macrophage cytosol requires the permeabilization of the phagosomal membrane by the three type VII secretion systems ESX-1, ESX-2, and ESX-4. TNT access to the cytosol results in NAD(P)^+^ depletion and activation of necroptotic cell death as shown previously^7^. Figure was created using BioRender.com.

The complete absence of surface-exposed CpnT in *esx-4*-deficient Mtb demonstrates that no other ESX system can substitute for ESX-4 in CpnT export. Our observation that the ESX-4 system also participates in the export of the pore-forming EsxE-EsxF complex to the outer membrane (Fig. 3), which was recently identified as an essential component of CpnT export^8^, further supports the important role of the ESX-4 system in TNT secretion by Mtb. In this regard, it is striking that the *esx-4* genes are co-regulated with the *cpnT* operon by the alternative sigma factor SigM^41^. Overexpression of the *sigM* gene induces transcription of the ESX-4 genes *esxU* and *esxT* by 1800- and 70-fold, respectively, and of the *esxF* and *esxE* genes by 60- and 100-fold, respectively^41^. These results reveal intricate functional and regulatory links between the ESX-4 system and CpnT. We propose that the ESX-4 core proteins EccB4, EccC4, EccD, and MycP4 provide the inner membrane component of the CpnT translocation system, while the EsxEF complex constitutes the outer membrane pore as previously suggested^8^ (Fig. 6). The ESX-4–EsxE-EsxF translocation system might not only translocate CpnT across the inner membrane but may also enable integration of CpnT in the outer membrane of Mtb. Although the Sec system translocates outer membrane proteins such as the Msp porins across the inner membrane in *M. smegmatis*^42^ in a similar manner as in Gram-negative bacteria^43^, protein integration into outer membranes in mycobacteria has been a glaring knowledge gap for more than two decades^44, 45^, as homologs of the β-barrel assembly machinery (Bam) of Gram-negative bacteria^46, 47^ are unknown in mycobacteria. Thus, it is conceivable that ESX-associated proteins such as EsxE–EsxF assist in integrating proteins in the outer membrane and participate in the function of this fascinating toxin secretion system.

### The ESX-1, ESX-2, and ESX-4 secretion systems are required for phagosomal permeabilization and protein trafficking into the cytosol of macrophages infected with *M. tuberculosis*

In our quest to understand how TNT is secreted and gains access to the cytosol of macrophages infected with Mtb, we showed that TNT trafficking to the cytosol requires a functional ESX-2 system in addition to the ESX-1 system (Fig. 4). Experiments with antibodies against Mtb proteins surprisingly revealed that the ESX-4 system is also required to permeabilize the phagosomal membrane in addition to ESX-1 and ESX-2 (Fig. 5a). These novel roles of the ESX-2 and ESX-4 systems in Mtb in phagosome rupture are consistent with the absence or largely reduced membrane damage caused by the *esx-2* and *esx-4*-deficient mutants compared to wt Mtb (Fig. 5d). Although the requirement of ESX-1 for phagosome rupture and egress of Mtb to the cytosol of infected macrophages has been described in numerous studies, it was believed until now, that ESX-1 is the only ESX system required for phagosomal permeabilization by Mtb^48, 49^. Our results challenge this paradigm and show that ESX-1 is essential, but not sufficient for the trafficking of Mtb proteins from the phagosome to the cytosol of infected macrophages. Instead, the combined activities of three ESX systems are required to efficiently rupture the phagosome. The ESX-2 system is restricted to Mtb and has not been previously studied to our knowledge. We hypothesize that the essential contribution of ESX-2 provides additional control of phagosomal permeabilization and bacterial escape in Mtb, which is not found in other bacteria. This process may have evolved in Mtb because translocation to the cytosol of infected macrophages not only provides access to essential nutrients such as carbon sources and iron and enables bacterial dissemination, but it also alerts the host immune system, e.g., by activating type I interferon production and the anti-bacterial selective autophagy pathway through the cytosolic DNA sensor cGAS^50^. Similarly, the ESX-4 system of the fast-growing *Mycobacterium abscessus* is involved in phagosomal damage and is required for growth in amoebae^17^. As *M. abscessus* only has the ESX-3 and ESX-4 systems, it has been proposed that its ESX-4 system operates as a surrogate of the ESX-1 and ESX-5 systems of Mtb^17^. In contrast, the ESX-4 system of *M. marinum* was not required for phagosomal membrane rupture and bacterial escape into the cytosol of infected macrophages^39^. These results underline that ESX systems can have divergent molecular functions in different mycobacterial species.

It is clear now that permeabilization of the phagosomal membranes by Mtb is a much more complex process than previously known, involving three ESX systems, the PDIM lipids^25, 30, 31^, and the host phospholipase A2^51^. How this process is regulated and how the different ESX systems interact with each other to permeabilize the phagosomal membrane is an exciting topic for future research.

### Model of toxin secretion and trafficking by *M. tuberculosis*

Based on the identification of ESX-4 as the sole type VII secretion system required for CpnT export in Mtb in this study and the essential role of the EsxEF proteins in this process as described in our recent report^8^, we propose the following model for TNT production, export, secretion, and trafficking by Mtb (Fig. 6). After inhalation, Mtb is engulfed by host alveolar macrophages, trapped inside the phagosome, and starts to replicate^52^. The alternative sigma factor SigM is induced in the late stationary phase^53^ and drives expression of the *esx-4* and *cpnT* operon genes^41^. Then, the ESX-4 core proteins EccB4, EccC4, EccD4, MycP4 assemble in the inner membrane, probably in a similar manner as shown for the ESX-3^54^ and ESX-5 systems^55^. After transcription of the *esxF* and *esxE* genes, the corresponding proteins form a complex and are translocated across the inner membrane in a process facilitated by the ESX-4 system. While we showed that EsxE and EsxF form a dimer^8^, it is unclear whether dimer formation precedes translocation as shown for their homologs ESAT-6 and CFP-10^56^. The EsxE-EsxF dimer forms a pore complex and integrates into the outer membrane^8^. The CpnT polypeptide is recognized by the ESX-4 system possibly via the ESX Y1 motif and translocated across the inner membrane. It should be noted that our data do not provide any insight into the folding state of CpnT or EsxE and EsxF during transport across the inner membrane. In the last step, the TNT domain is translocated across the outer membrane and exposed on the cell surface with the help of the EsxE-EsxF pore and possibly with the involvement of the ESX-motif Y3 (Fig. 6). It is unclear whether CpnT is secreted as a full-length protein, e.g., integrated into extracellular membrane vesicles^57, 58^, or whether its C-terminal domain TNT is cleaved off and released from the cell surface as proposed previously^5^. At this stage, CpnT/TNT is still trapped inside the phagosome as can be observed in Fig. 4a. The concerted activities of the ESX-1, ESX-2, and ESX-4 systems are required to permeabilize the phagosomal membrane and enable trafficking of the toxin into the cytosol of infected macrophages, where TNT hydrolyzes NAD^+^^6^. When NAD^+^ levels drop below a certain threshold, RIPK3 is activated and induces a programmed cell death called necroptosis^7^. This leads to further membrane damage enabling Mtb to leave the phagosome^10^. The subsequent escape from the destroyed macrophage is the first step for Mtb to disseminate in the infected host.

This study shows that export of the outer membrane protein CpnT and phagosomal rupture are the first known molecular functions of the ESX-4 system in Mtb, highlighting the central role of ESX-4 in CpnT function and Mtb cytotoxicity. The paradigm-changing discovery that both the ESX-2 and ESX-4 systems need to act in concert with the ESX-1 system to permeabilize the phagosomal membrane raises important questions regarding the molecular mechanism of this process and the regulation of these activities. This study presents a major advancement in our understanding of protein secretion and trafficking by Mtb and will certainly stimulate further research in these important areas of Mtb biology.

## Methods

### Bacterial strains and culture conditions

*E. coli* MachI cells (Thermo Fisher Scientific) used for cloning experiments were routinely grown in Luria-Bertani agar/broth at 37 °C and supplemented with kanamycin (30 µg/ml) and hygromycin (200 µg/ml) when required. The auxotrophic Mtb mc^2^6206 and derivative strains were grown in Middlebrook 7H9 or on 7H10 (Difco) agar plates supplemented with 10% Albumin Dextrose Sodium (ADS; 8.5 g/L NaCl, 20 g/L dextrose, 50 g/L bovine serum albumin fraction V), 0.5% glycerol, 0.2% casamino acids, 0.02% tyloxapol, 24 µg/ml pantothenate, and 50 µg/ml l-leucine. Mtb Erdman and derivative strains were grown in the same media as Mtb mc^2^6206 but without leucine and pantothenate. For the Mtb cultures, kanamycin (30 µg/ml), hygromycin (50 µg/ml), and ATc (100 ng/ml) were used at the indicated concentrations when required. All bacterial strains used are shown the Supplementary Table 1. Mtb cultures were never passaged more than twice.

### Plasmid construction

All molecular cloning was performed using standard methods using restriction enzymes. KoD Hotstart DNA polymerase was purchased from Millipore. NEB Quick Ligase and restriction enzymes were purchased from New England Biolabs. *E. coli* MachI competent cells (Thermo Fisher Scientific) were used for all molecular cloning. The *esxF-esxE-cpnT-ift* operon was amplified from Mtb H37Rv genomic DNA (BEI Resources) using oligonucleotides containing an upstream PacI site and a downstream HindIII site. The resulting PCR product was purified by gel electrophoresis. The PCR fragments were then digested with PacI/HindIII, purified by gel electrophoresis, and ligated into the PacI/HindIII-digested pML3042 backbone using NEB Quick Ligase. Overlap two-step PCR was used to generate the Y1, Y2, and Y3 mutants. The final PCR fragment was digested and purified as described above. Ligation samples were then transformed into MachI *E. coli*. Colonies containing inserts were examined by restriction digestions and DNA sequencing using the UAB Heflin Genomics core facility. All plasmids and primers used are shown in Supplementary Tables 2 and 3, respectively.

### Deletion of the *eccC2* and *eccC4* genes in *M. tuberculosis*

The *eccC2* and *eccC4* gene deletion mutants were generated by homologous recombination as described previously^59, 60^. Specifically, the *eccC2* and *eccC4* upstream and downstream regions were amplified with the indicated primers (Supplementary Fig. 7, Supplementary Table 3) and cloned into pML2424 to construct pML4245 and pML4255 (Supplementary Fig. 7, Supplementary Table 2). These plasmids were used to create unmarked, in-frame deletions of 4191 bp and 3687 bp within the *eccC2* and *eccC4* genes, respectively, in the avirulent Mtb mc^2^6206 strain (Supplementary Fig. 7a–d). The parent Mtb mc^2^6206 strain harboring the *eccC2* deletion vector pML4245 or *eccC4* deletion vector pML4255 were grown on selective 7H10 plates containing 10% ADS, 0.5% glycerol, 24 μg/mL pantothenate, 50 μg/mL l-leucine, 0.2% casamino acids, 2% (w/v) sucrose and 50 μg/mL hygromycin. The plates were cultured at 40 °C for 4–5 weeks to select for double cross-over (DCO) clones, which were then examined by colony PCR. Competent cells of the validated DCO mutants were transformed with the temperature-sensitive plasmid pML2714 expressing Cre recombinase to remove the *loxP-*flanked *gfp2*^*+*^_*m*_*-hyg* cassette on 7H10 plates with 30 μg/mL kanamycin at 37 °C. Clones were then validated for loss of GFP and RFP was examined by flow cytometry using the marked strain as a control. Positive clones were then incubated at 40 °C to remove pML2714. The final unmarked Mtb *eccC2* and *eccC4* deletion strains (Δ*esx-2* and Δ*esx-4* strains, respectively) were validated by PCR (Supplementary Fig. 7b, d). The presence of PDIMs, which are essential for Mtb infection and can be spontaneously lost during mutant construction^61^, was confirmed using a previously published protocol^62^. A single colony was inoculated in 10 mL of medium and grown to late log phase and used to make seed stocks for further experiments.

### Culture and differentiation of THP-1 monocytes

Human THP-1 monocytes (ATCC TIB-202) were propagated in RPMI-1640 (HyClone) supplemented with 10% FBS (Gibco), 10 mM HEPES (HyClone), 2 mM l-glutamine (HyClone), 1× non-essential aminoacids (HyClone), 100 U/ml penicillin (Gibco), 100 μg/ml streptomycin (Gibco) and 250 ng/ml amphotericin B (Gibco). The day prior to infection, THP-1 monocytes were seeded on sterile coverslips in 24-well plates and differentiated for 24 h into macrophage-like cells with 50 ng/mL 12-phorbol 13-myristate acetate (Sigma). Cells were grown in a 37 °C humidified incubator at 5% CO_2_. Cell cultures were routinely tested for mycoplasma contamination as described elsewhere^63^.

### Infection of THP-1 macrophages with *M. tuberculosis*

The infection of differentiated THP-1 macrophages by Mtb was performed as we previously described^7^. In brief, Mtb cells were harvested in mid-log phase (OD_600_ of 0.6). THP-1 macrophages were infected with the Mtb strains at an MOI of 10:1 for 4 h. Macrophage monolayers were incubated with a medium containing 20 mg/mL gentamycin for 1 h to kill extracellular bacteria and then kept in a medium without antibiotics for a total of 48 h.

### Immunoblotting

Whole-cell lysates and culture filtrates were obtained from *M. tuberculosis* cultures. In brief, bacteria were pelleted, washed three times with PBS, resuspended in PBS containing EDTA-free protease inhibitors (Thermo Scientific), and subjected to bead beating to lyse the cells (whole-cell lysate). The culture supernatants were sequentially filtered through 0.45-µm and 0.22-µm syringe filters (Millipore), centrifuged (4000 × *g*, 15 min, 4 °C) to remove the remaining cellular debris, and then concentrated to ~100-fold using 3-kDa-cutoff Amicon Ultra centrifugal filtration devices (Millipore). Then, 50 μg of whole-cell lysates or 15 μg of culture filtrates were used for immunoblotting for detection of Mtb proteins. Samples were prepared with Laemmli protein loading dye, run on a 10% sodium dodecyl sulfate (SDS)-polyacrylamide gel and transferred to a polyvinylidene difluoride membrane with the Trans-Blot Turbo Transfer System (Bio-Rad). Blots were blocked with 5% skim milk (BD) in TBS + 1% Tween20 (TBST), washing three times with TBST between detection steps. Primary and secondary antibody solutions were prepared in TBST + with 2.5% skim milk. Primary antibody detection was performed using rabbit polyclonal anti-TNT (1:1000), rabbit polyclonal anti-IFT (1:1000), rabbit polyclonal anti-PPE41 (1:250), rabbit polyclonal anti-EccD_5_ (1:1000), mouse monoclonal anti-LpqH (1:1000, BEI Resources), rabbit polyclonal anti-GlpX (1:1000, BEI Resources), rabbit polyclonal anti-CFP-10 (1:1000, BEI Resources), rabbit polyclonal anti-Ag85 (1:1000, BEI Resources), and mouse monoclonal anti-MctB (1:1000). Secondary detection was performed with donkey anti-rabbit IgG-IRDye 680RD or goat anti-mouse IgG-IRDye 800CW at a dilution of 1:5000 (LI-COR biosciences). All the antibodies used in this study are listed in Supplementary Table 4. Western blots were imaged using a Li-Cor Odyssey.

### Subcellular fractionation of *M. tuberculosis*

Mtb mc^2^6206 and derivative mutant and complemented strains were grown to an OD_600_ of 2. Cells were harvested by centrifugation, washed twice with PBS containing 1 mM phenylmethylsulfonyl (PMSF) and lysed by sonication (30 min, 42 Watt, 30 sec on/off) in PBS + PMSF. Cell debris was removed from the lysate by centrifugation at 2000 × *g* for 10 min at 4 °C, and the supernatant was centrifuged at 100,000 × *g* for 1 h at 4 °C. The supernatant (C1) was transferred to a separate tube, and the pellet was resuspended in the same volume of PBS + PMSF as C1 and designated M1. Both C1 and M1 fractions were centrifuged at 100,000 × *g* for 1 h at 4 °C. The supernatant containing the cytosolic fraction was transferred to a new tube and labeled C2, and the membrane pellet fraction was resuspended in the same volume of PBS + PMSF + 1% SDS as that used for C2 and designated M2. Proteins in M2 (membrane fraction) and C2 (soluble fraction) were detected by Western blots as described above. The membrane control protein MctB and the cytosolic control protein GlpX were detected by specific mouse monoclonal and rabbit polyclonal antibodies, respectively.

### Surface detection of CpnT in *M. tuberculosis* by flow cytometry

Mtb mc^2^6206 and derivative mutant and complemented strains were used for surface detection experiments. All the steps of the staining procedure were performed at room temperature and included three washes with PBS between each step. Cultures were grown to an OD_600_ of 2 and fixed with 4% paraformaldehyde for 15 min. Cells were blocked with 5% normal goat serum for 30 min and incubated with 1:200 anti-TNT antibody for 1 h. Cells were then incubated with 1:300 Alexa Fluor-488 goat anti-rabbit IgG (H+L) for 1 h and analyzed via flow cytometry on a BD Accuri 6 cytometer. Since we worked with pure cultures of Mtb derived from a single colony with identical cells, we did not use gating procedures to include all detected particles in our measurements. Data were analyzed by FlowJo v10. All samples were run with acquisition limits of 50,000 events or 150 µl. Surface-accessible CpnT was quantified by measuring fluorescence and displayed as histograms.

### Surface detection of CpnT in *M. tuberculosis* by fluorescence microscopy

Mtb cultures were grown to an OD_600_ of 0.6, and a 1 ml aliquot was incubated with 100 μg/ml DMN-trehalose at 37 °C for one doubling time (24 h) on an end-over-end rotor to label the outer membrane of metabolically active Mtb cells^64^. In some experiments, the Mtb membrane was visualized by labeling with FM-464x (Thermo Fisher Scientific) at a concentration of 5 µg/ml overnight at 37 °C. All following the steps of the staining procedure were performed at room temperature and included three washes with PBS between each step. The stained cells were fixed with 4% paraformaldehyde (Electron Microscopy Sciences) in PBS for 20 min, blocked with 2.5% normal goat serum (Thermo Fisher Scientific) for 30 min and then incubated with 1:50 anti-TNT antibody for 1 h. Cells were then incubated with 1:100 goat anti-rabbit Alexa Fluor-594 or Alexa Fluor-488 for 1 h and imaged on agarose pads. Images were taken using a Zeiss Axiovert 200 epifluorescent microscope with a ×100/1.4 Plan apochromat coupled to a Zeiss Axiocam MRc camera (Carl Zeiss, Thornwood, NY). Images were collected using Axiovision v4.5 software and analyzed further in ImageJ v1.8.0_172 to create composites.

### Fluorescence microscopy of macrophages infected with *M. tuberculosis*

For the infection experiments, Mtb cells were stained with DMN-Trehalose as described above^64^ and were used to infect THP-1 macrophages on glass coverslips for 48 h. The staining procedure was performed at room temperature as previously described^65^ with modifications. In brief, macrophages were fixed with 3% paraformaldehyde in PBS for 10 min and subsequently quenched with 10 mM ammonium chloride in PBS for 10 min, followed by three washes with PBS. Cells were permeabilized with 25 μg/mL digitonin in PBS alone for 5 min (permeabilization of the plasma membrane only) or in combination with 0.2% Triton X-100 in PBS for an additional 5 min (permeabilization of plasma and phagosomal membranes), as we did previously^6^. Cells were then washed three times with PBS and blocked with 2.5% normal goat serum (Thermo Fisher Scientific) in PBS for 10 min. Thereafter, cells were incubated with the rabbit polyclonal antibodies anti-TNT (1:100), anti-Mtb (1:200, Abcam), anti-Galectin-3 (1:100, Abcam) or mouse monoclonal anti-Ag85 antibody (1:100, BEI Resources) for 60 min. After three washes with PBS, cells were incubated with secondary anti-rabbit or anti-mouse Alexa Fluor-594 (1:300, Thermo Scientific) for 60 min. Nuclei were stained with DAPI (Thermo Scientific), following the manufacturer’s instructions. Coverslips were mounted onto microscope slides with ProLong Glass Antifade Mounting solution (Invitrogen) for fluorescence imaging. Images were taken using a Zeiss Axiovert 200 epifluorescent microscope with a ×100/1.4 Plan apochromat coupled to a Zeiss Axiocam MRc camera (Carl Zeiss, Thornwood, NY). Images were collected using Axiovision v4.5 software and analyzed further in ImageJ v1.8.0_172 to create composites.

### Macrophage viability

The cell viability of macrophages was assessed as described previously^7^ with the Cell-Titer Glo 2.0 Luminescent Viability Assay (Promega), a luciferase-based assay that determines the number of viable cells in culture by quantifying ATP. Signal was recorded with a Cytation 3 plate reader (Biotek) and analyzed with Gen5 software v3.0.

### Statistical analysis

Graphpad Prism v6 or SigmaPlot v11.0 (Systat Software) were used for creating plots and for statistical analysis. Data are presented as mean ± SEM of at least three independent experiments unless otherwise indicated; *p* values were calculated using one-way analysis of variance with Dunnett’s correction, and *p* < 0.05 was defined as significant. All figures were created with Coreldraw v17 or BioRender.

### Reporting summary

Further information on research design is available in the Nature Research Reporting Summary linked to this article.

## Supplementary information

Supplementary Information

Reporting Summary

## Data Availability

The data generated in this study are provided in the Supplementary Information/Source Data file. Any additional data are available upon request. Source data are provided with this paper.

## Source data

Source Data
