# Supplementary material for: Toxin secretion and trafficking by Mycobacterium tuberculosis
Source: Nat Commun. 2021 Nov 15;12:6592. doi: 10.1038/s41467-021-26925-1 (PMC8593097; doi:10.1038/s41467-021-26925-1)
Supplement: Supplementary file 1 — Supplementary Information [file 41467_2021_26925_MOESM1_ESM.pdf]

SUPPLEMENTARY INFORMATION

Toxin secretion and trafficking by *Mycobacterium tuberculosis*

David Pajuelo[a], Uday Tak[a,&], Lei Zhang[a], Olga Danilchanka[a,$], Anna D. Tischler[b] and Michael Niederweis[a*]

[a] Department of Microbiology, University of Alabama at Birmingham, 609 Bevill Biomedical Research Building, 845 19th Street South, Birmingham, AL 35294, USA

[b] Department of Microbiology and Immunology, University of Minnesota Twin Cities, Minneapolis, Minnesota, MN 55455, USA

[&] Current address: University of Colorado Boulder, Jennie Smoly Caruthers Biotechnology Building B255, 3415 Colorado Avenue, Boulder, Colorado 80303.

[$] Current address: Merck & Co., Inc., Cambridge, MA 02141, USA

*** For correspondence:**

E-Mail: mnieder@uab.edu

Phone: +1-205-975-4390

Supplementary Figures

a

```
>M. tuberculosis H37Rv|Rv3903c: 846 aa
MAPLAVDPAALDSAGGAVVAAGAGLGAVISSLTAALAGCAGMAGDDPAGAVFGRSYDGSA
AALVQAMSVARNGLCNLGDGVRMSAHNYSLAEAMSDVAGRAAPLPAPPPSGCVGVGAPPS
AVGGGGGAPKGWGWVAPYIGMIWPNGDSTKLRAAAVAWRSAGTQFALTEIQSTAGPMGVI
RAQQLPEAGLIESAFADAYASTTAVVGQCHQLAAQLDAYAARIDAVHAAVLDLLARICDP
LTGIKEVWEFLTDQDEDEIQRIAHDIAVVVDQFSGEVDALAAEITAVVSHAEAVITAMAD
HAGKQWDRFLHSNPVGVVIDGTGQQLKGFGEEAFGMAKDSWDLGPLRASIDPFGWYRSWE
EMLTGMAPLAGLGGENAPGVVESWKQFGKSLIHWDEWTTNPNEALGKTVFDAATLALPGG
PLSKLGSKGRDILAGVRGLKERLEPTTPHLEPPATPPRPGPQPPRIEPPESGHPAPAPAA
KPAPVPANGPLPHSPTESKPPPVDRPAEPVAPSSASAGQPRVSAATTPGTHVPHGLPQPG
EHVPAQAPPATTLLGGPPVESAPATAHQPQWATTPAAPAAAPHSTPGGVHSTESGPHGRS
LSAHGSEPTHDGASHGSGHGSGSEPPGLHAPHREQQLAMHSNEPAGEGWHRLSDEAVDPQ
YGEPLSRHWDFTDNPADRSRINPVVAQLMEDPNAPFGRDPQGQPYTQERYQERFNSVGPW
GQQYSNFPPNNGAVPGTRIAYTNLEKFLSDYGPQLDRIGGDQGKYLAIMEHGRPASWEQR
ALHVTSLRDPYHAYTIDWLPEGWFIEVSEVAPGCGQPGGSIQVRIFDHQNEMRKVEELIR
RGVLRQ
```

Channel domain NAD$^+$ Glycohydrolase domain **Putative ESX Motif**

b

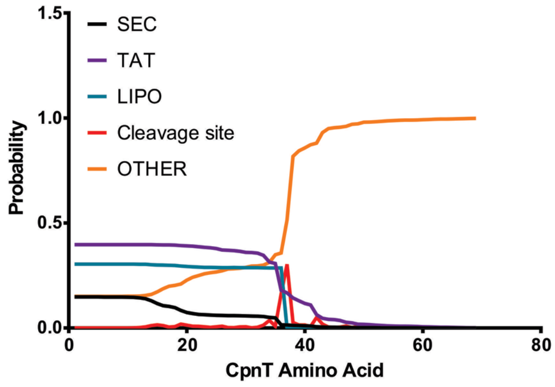

c

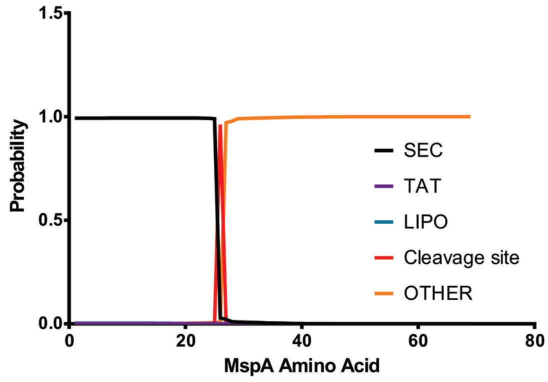

Supplementary Figure 1. Bioinformatic analysis of CpnT.

a. Amino acid sequence of CpnT (Rv3903c) of *Mycobacterium tuberculosis.* Analysis of the N-terminal amino acid sequences of CpnT (**b**) and the *Mycobacterium smegmatis* porin A (MspA) (**c**), which has a verified SEC signal sequence, using the SignalP 5.0 server. SEC = general secretory pathway signal, TAT = twin arginine transport pathway, LIPO = lipoprotein, Other = probability of having no known signal peptide.

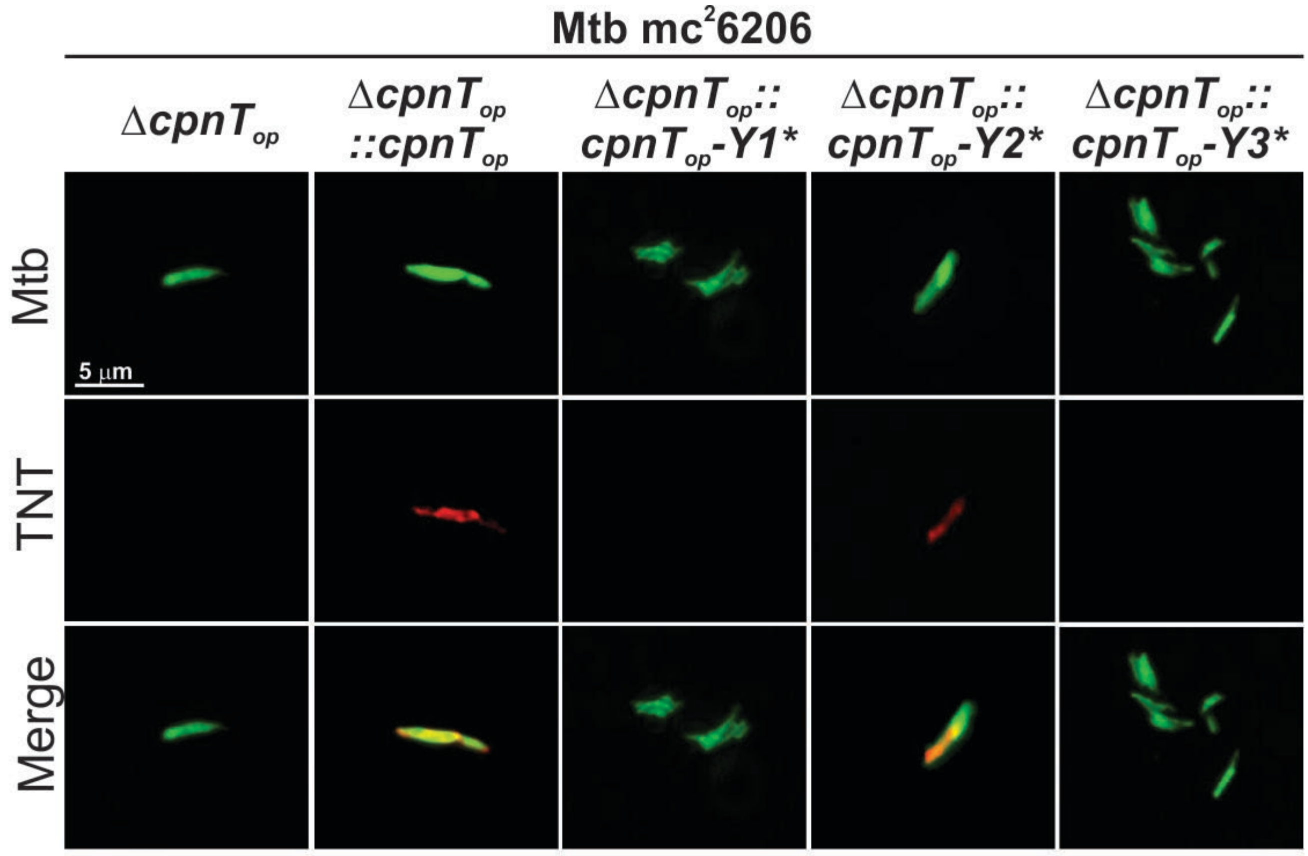

Supplementary Figure 2. The ESX Y1 and Y3 motifs are required for surface accessibility of CpnT in *M. tuberculosis*.

Full data set for Figure 1c with each fluorescence channel shown separately. The indicated Mtb strains were labeled with the metabolic dye DMN-Trehalose (green) and surface-exposed CpnT was detected using an anti-TNT and Alexa Fluor-594 antibodies (red). Shown are representative images from at least two independent experiments (n≥2).

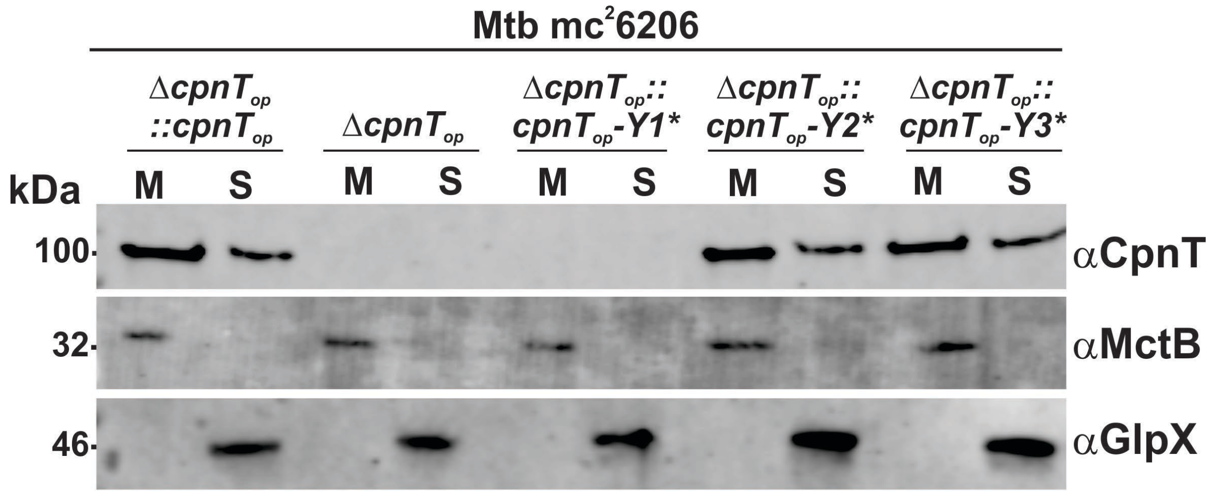

Supplementary Figure 3. Subcellular localization of CpnT with ESX motif mutations in *M. tuberculosis*.

The cell lysates of the indicated Mtb strains were separated in water-soluble (S) and membrane (M) fractions by ultracentrifugation. The Δ$cpnT_{op}$ strain was transformed with a plasmid encoding the wt CpnT operon (pML3009), the CpnT operon with the Y1* mutation (pML3966), Y2* mutation (pML3967) or the Y3* mutation (pML3968). The localization of CpnT in these fractions was determined by immunoblots using an anti-TNT antibody and the proteins MctB (membrane; M) and GlpX (water-soluble; S) as markers, with their respective antibodies. Shown are representative blots from three independent experiments (n=3). Source data are provided in the Source Data file.

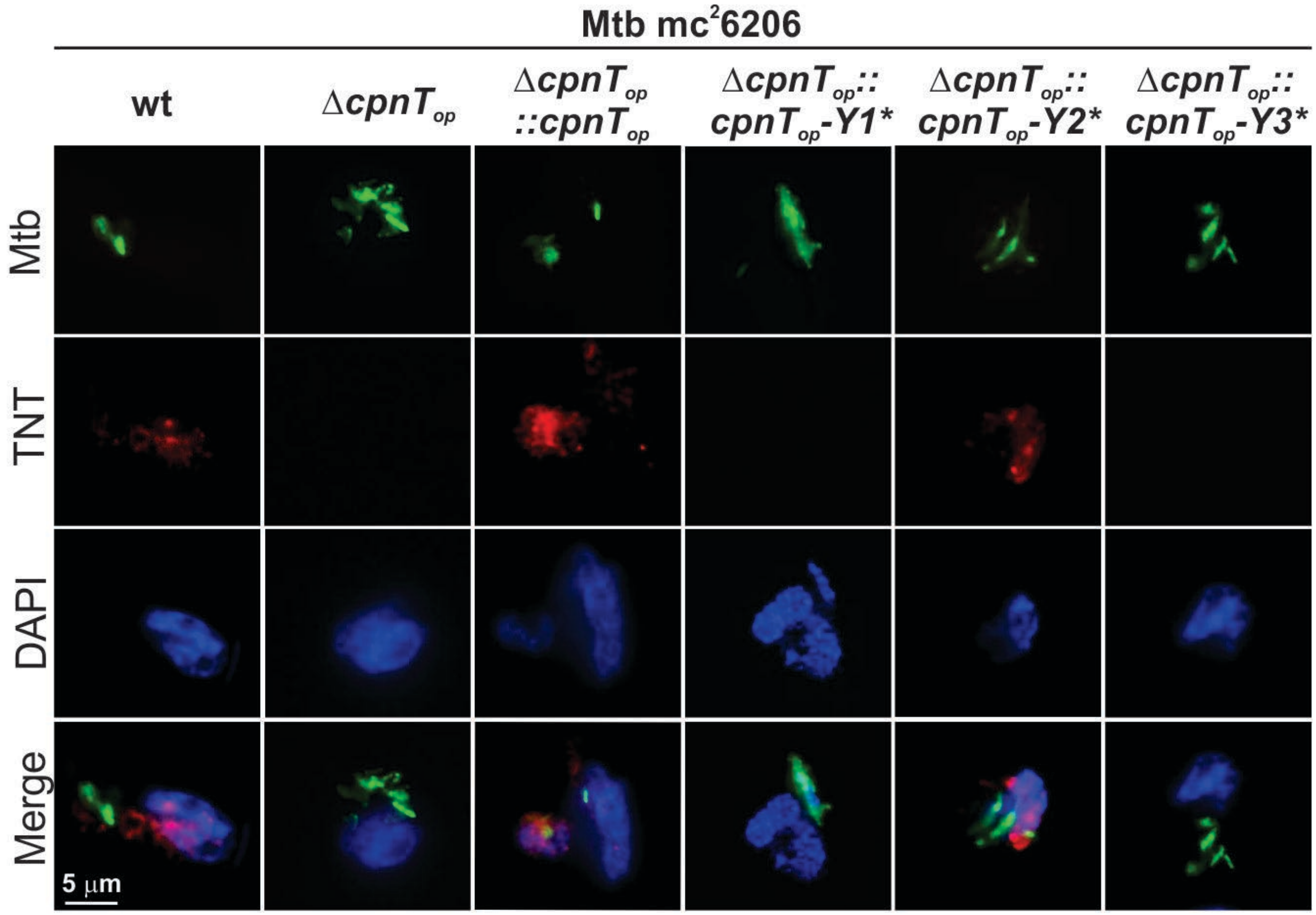

Supplementary Figure 4. CpnT with mutated Y1 and Y3 ESX motifs does not reach the cytosol of macrophages infected with *M. tuberculosis.*

Full data set for Figure 1e with each fluorescence channel shown separately. Secretion of TNT into the cytosol of Mtb-infected macrophages. The indicated Mtb strains were labelled with the metabolic dye DMN-Trehalose (green) and used to infect THP-1 macrophages at a MOI of 10:1. The macrophages were permeabilized with Triton X-100 48 h after infection, stained with an anti-TNT antibody and Alexa Fluor-594 secondary antibody (red). The macrophage nuclei were stained with DAPI. Shown are representative images from at least two independent experiments (n≥2).

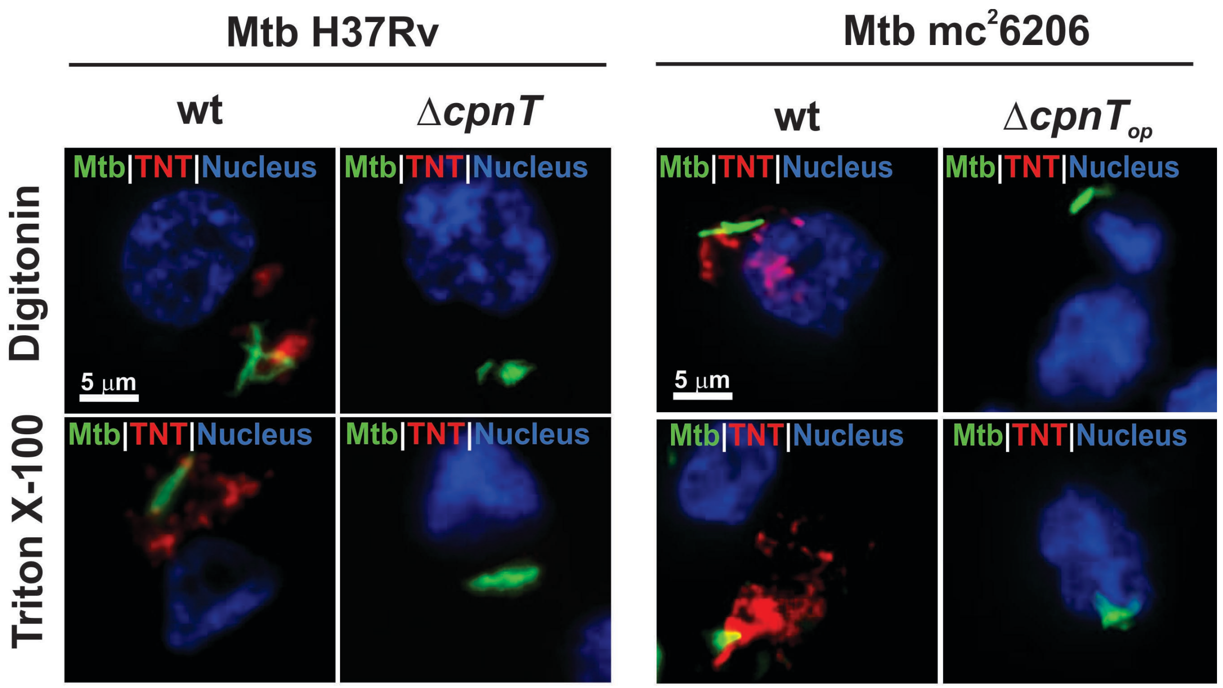

Supplementary Figure 5. TNT secretion in macrophages infected with virulent Mtb H37Rv and avirulent Mtb mc^{2}6206.

The indicated Mtb strains were labelled with the metabolic dye DMN-Trehalose (green) and used to infect THP-1 macrophages at a MOI of 10:1. 48 h after infection, the macrophages were fixed with 4% paraformaldehyde (H37Rv 1h; mc^{2}6206 10 min), then permeabilized with digitonin to enable access of antibodies to the cytoplasm, or with Triton X-100 for access to intracellular compartments. Then, cells were stained with an anti-TNT and with an Alexa Fluor-594 secondary antibody (red). The macrophage nuclei were stained with DAPI. Images were taken from Figure 4a (mc^{2}6026) and Figure 4e (H37Rv). Shown are representative images from at least two independent experiments (n≥2).

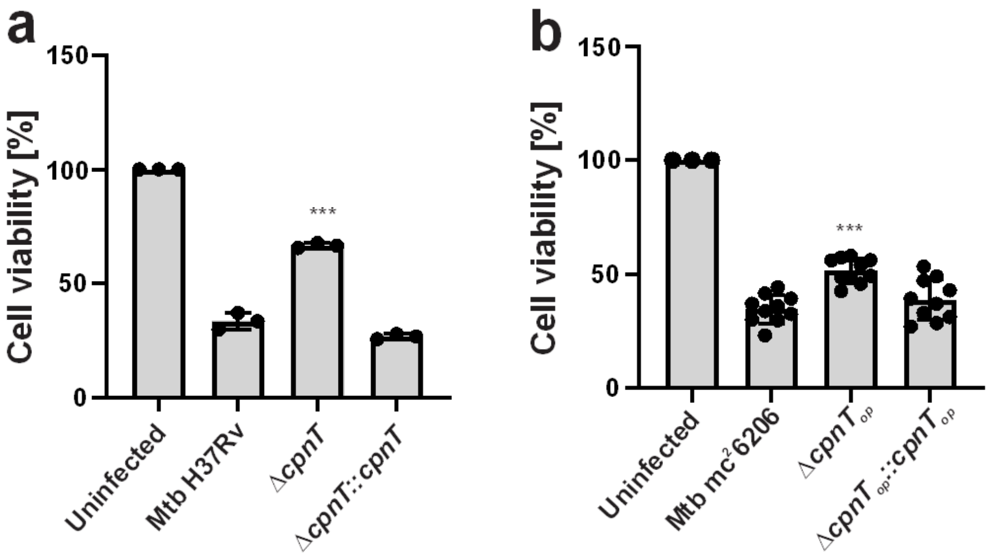

Supplementary Figure 6. Cytotoxicity of virulent Mtb H37Rv and avirulent Mtb mc^{2}6206 in infected macrophages.

Cell viability of Mtb-infected macrophages was measured as the total ATP content with a luminescent ATP detection assay kit. Data are represented as mean ± SEM. Asterisks indicate significant differences (p-value<0.05, calculated using the One-way ANOVA with Dunnett's correction) compared with the virulent Mtb H37Rv (**a**) and the avirulent Mtb mc^{2}6206 strain (**b**). The mc^{2}6026 data were taken from Figure 4d. Data are represented as mean ± SEM of at least two independent experiments (n≥2). Source data are provided in the Source Data file.

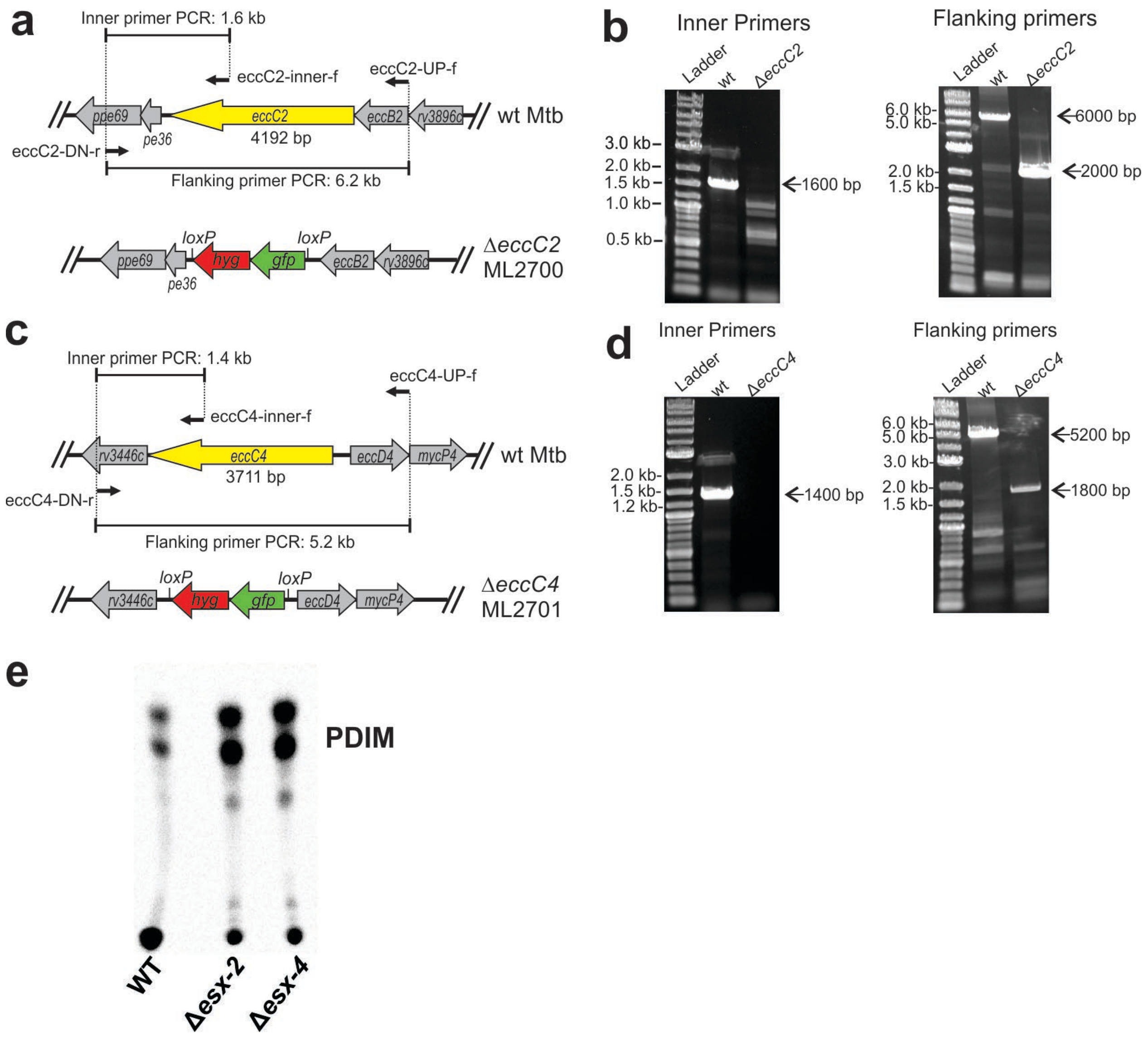

Supplementary Figure 7. Construction and validation of the *M. tuberculosis* Δ*eccC2* and Δ*eccC4* strains.

a. Upstream and downstream regions of *eccC2* and binding sites for primers with the indicated PCR fragment size for each primer pair. **b.** PCR validation of the *eccC2* unmarked deletion mutant ML2691 (Supplementary Table 1) using inner and flanking primers (Supplementary Table 4). **c.** Upstream and downstream regions of *eccC4* and binding sites for primers with the indicated PCR fragment size for each primer pair. **d.** PCR validation of the *eccC4* unmarked deletion mutant ML2690 (Supplementary Table 1) using inner and flanking primers (Supplementary Table 4). **e.** Thin layer chromatography of total lipids extracted from ^{14}C-propionate-labeled Mtb strains indicating the presence of the outer membrane lipid PDIM.

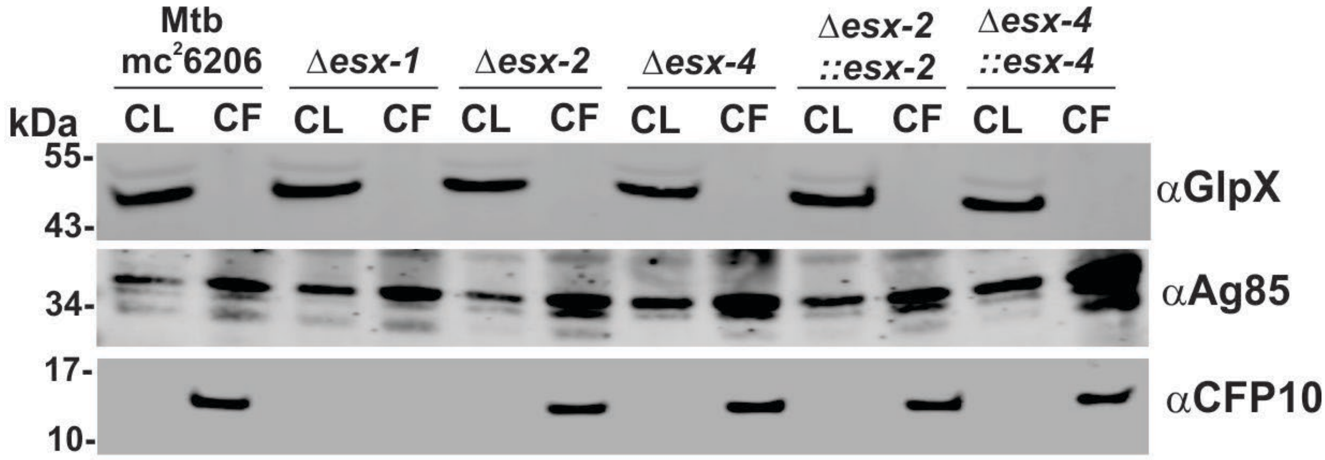

Supplementary Figure 8. The activity of the ESX-1 system is not impaired in the *esx-2 and esx-4* deletion mutants.

The activity of the ESX-1 system was analyzed by detecting the secreted ESX-1 substrate CFP-10 in cellular fractions of Mtb. Proteins from the whole cell lysates (CL) and culture filtrates (CF) of the indicated Mtb strains were analyzed by immunoblotting to detect CFP-10 with specific antibodies. Antibodies against GlpX and Ag85 were used as controls for the whole cell lysate and culture filtrate fractions, respectively. The Δ*esx-1* strain has a deletion of the RD1 region and lacks the *panCD* genes. Shown are representative blots from three independent experiments (n=3). Source data are provided in the Source Data file.

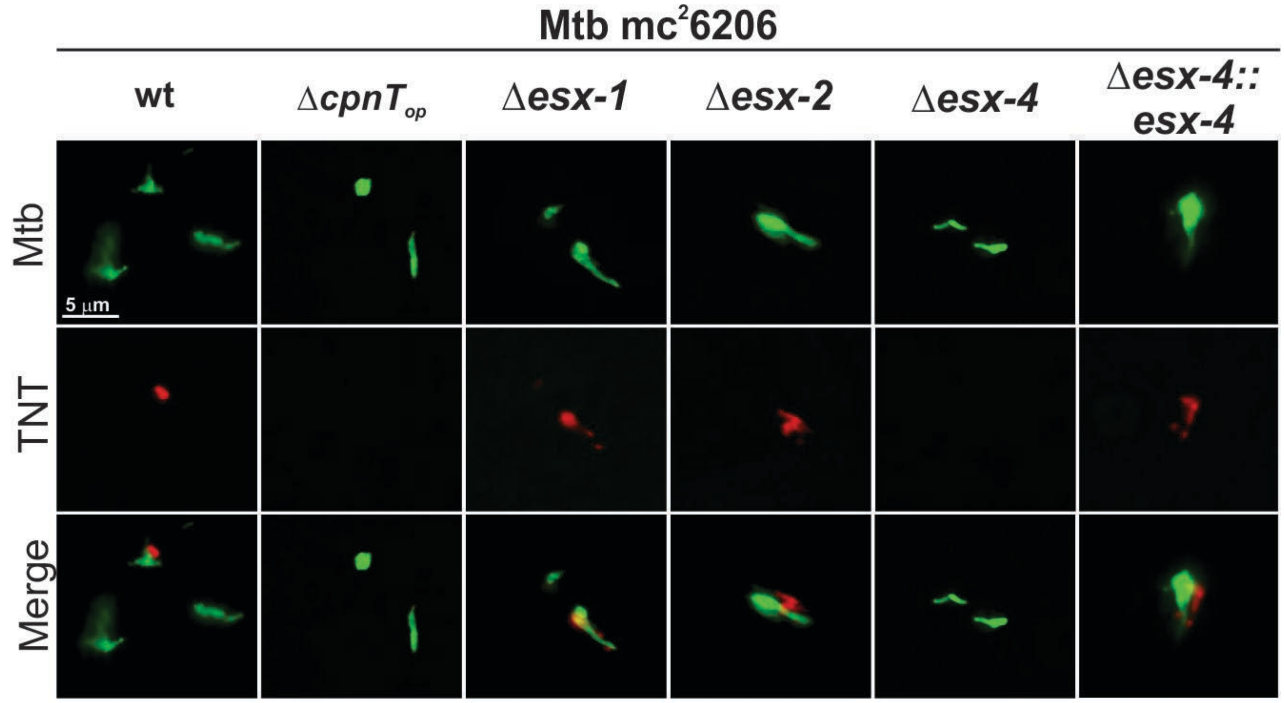

Supplementary Figure 9. The ESX-4 system is required for CpnT export and surface accessibility in *M. tuberculosis*.

Full data set for Figure 2a with each fluorescence channel shown separately. The indicated Mtb strains were labeled with the metabolic dye DMN-Trehalose (green) and surface-exposed CpnT was detected using an anti-TNT and Alexa Fluor-594 antibodies (red). The Δ*esx-1* strain has a deletion of the RD1 region and lacks the *panCD* genes. Shown are representative images from at least two independent experiments (n≥2).

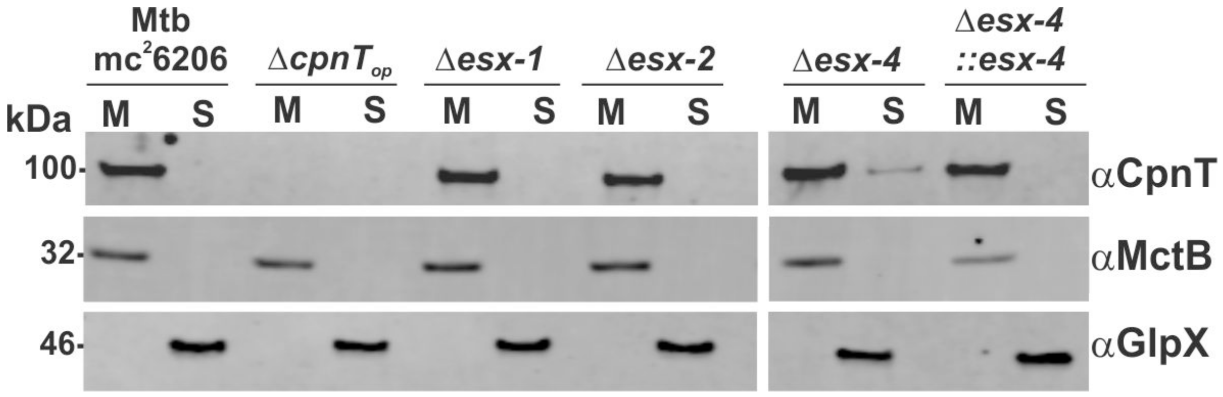

Supplementary Figure 10. Subcellular localization of CpnT in the *esx-1*, *esx-2* and *esx-4* deficient *M. tuberculosis* strains.

The cell lysates of the indicated Mtb strains were separated in membrane (M) and water-soluble (S) fractions by ultracentrifugation. The localization of CpnT in these fractions was determined by immunoblots using an anti-TNT antibody. The proteins MctB and GlpX were used as markers of the membrane and soluble fractions, respectively. The Δ*esx-1* strain has a deletion of the RD1 region and lacks the *panCD* genes. Shown are representative blots from three independent experiments (n=3). Source data are provided in the Source Data file.

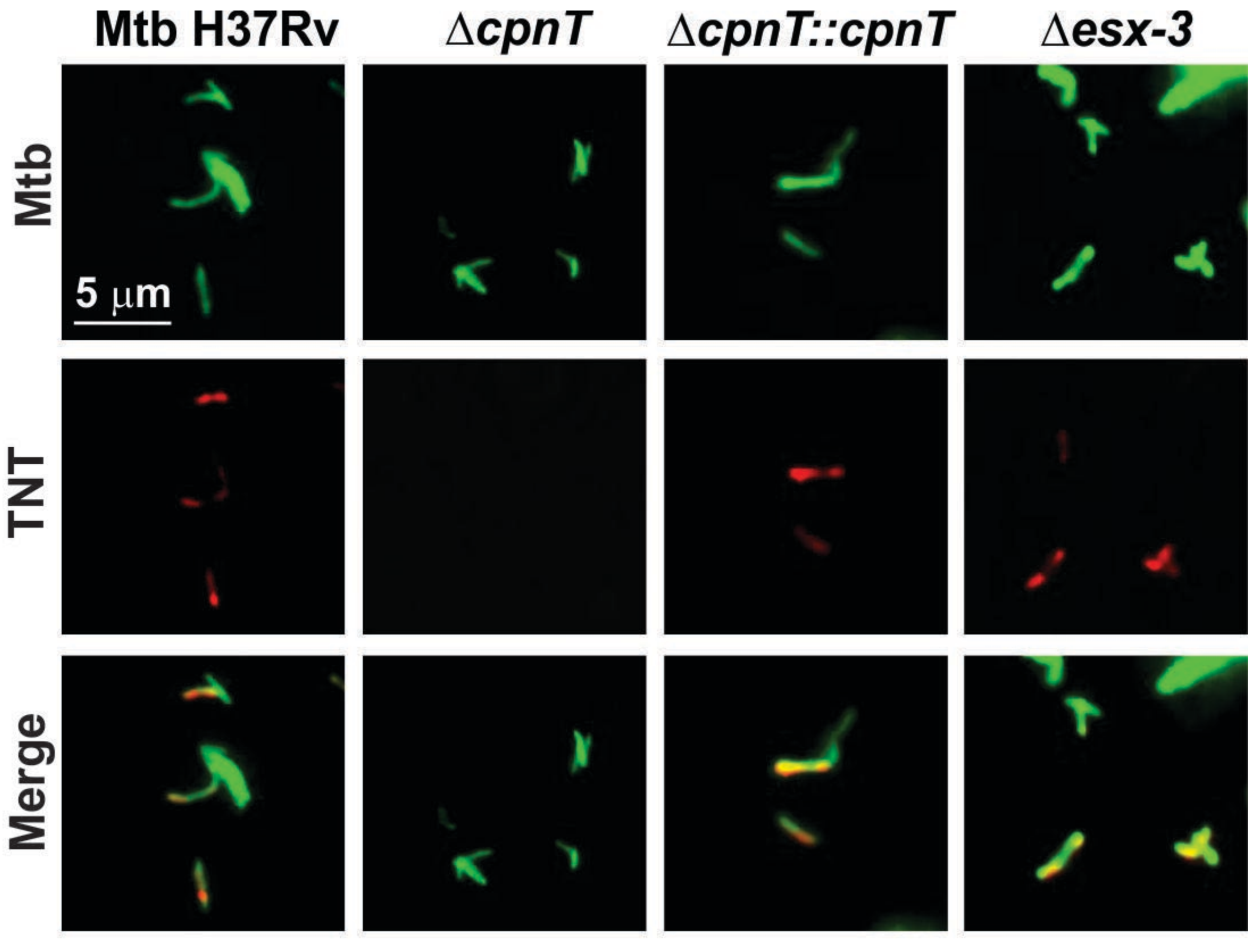

Supplementary Figure 11. The ESX-3 system does not participate in CpnT export in *M. tuberculosis*.

Full data set for Figure 2d with each fluorescence channel shown separately. The indicated Mtb strains were labeled with the metabolic dye DMN-Trehalose (green) and surface-exposed CpnT was detected using an anti-TNT and Alexa Fluor-594 antibodies (red). Shown are representative images from at least two independent experiments (n≥2).

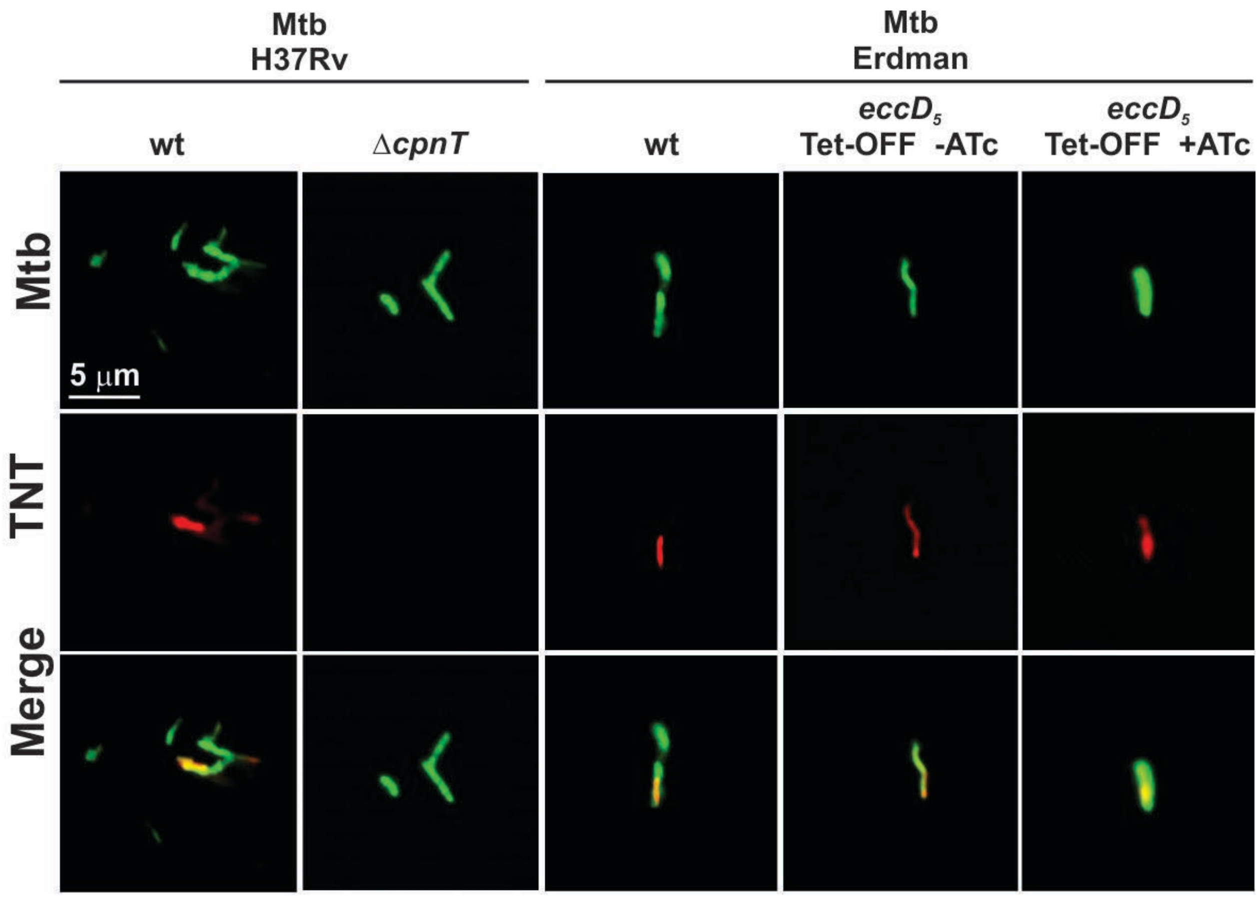

Supplementary Figure 12. The ESX-5 system does not participate in CpnT export in *M. tuberculosis*.

Full data set for Figure 2g with each fluorescence channel shown separately. The indicated Mtb strains were labeled with the metabolic dye DMN-Trehalose (green) and surface-exposed CpnT was detected using an anti-TNT and Alexa Fluor-594 antibodies (red). Shown are representative images from at least two independent experiments (n≥2).

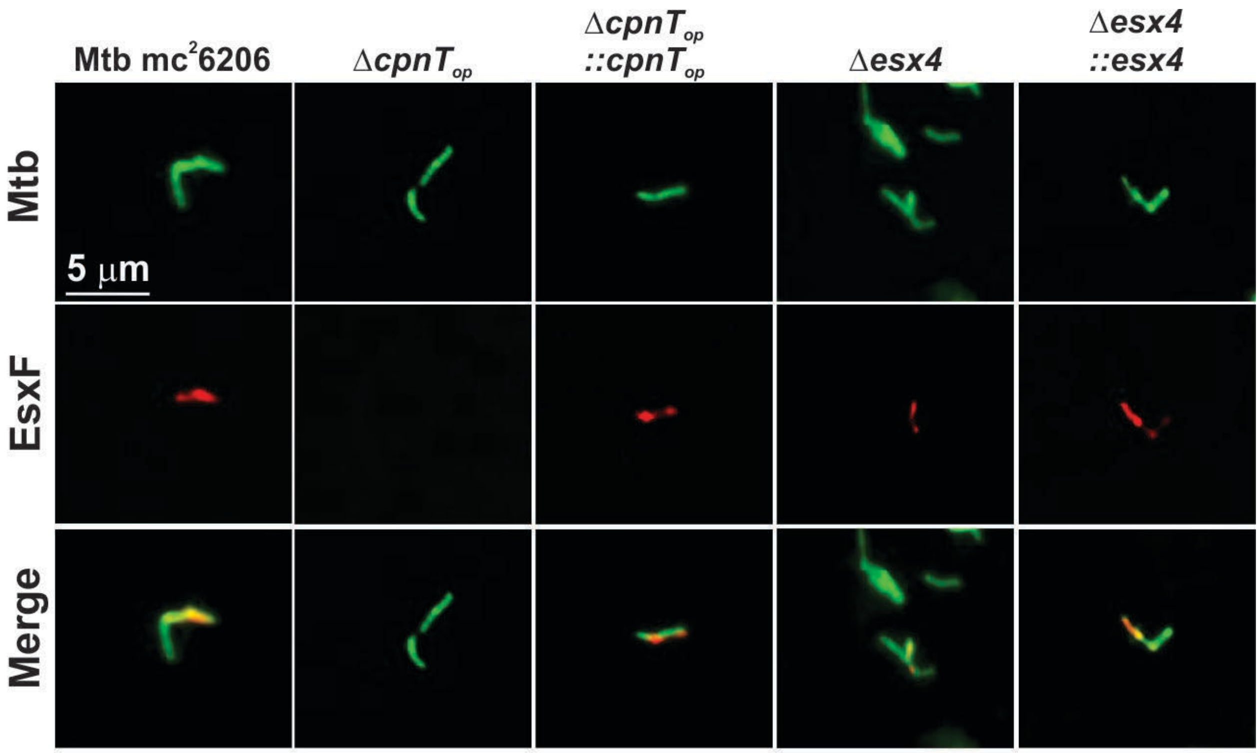

Supplementary Figure 13. The ESX-4 system is involved in EsxF export in *M. tuberculosis*.
Full data set for Figure 3a with each fluorescence channel shown separately. The indicated Mtb strains were labeled with the metabolic dye DMN-Trehalose (green) and surface-exposed CpnT was detected using an anti-TNT and Alexa Fluor-594 antibodies (red). Shown are representative images from three independent experiments (n=3).

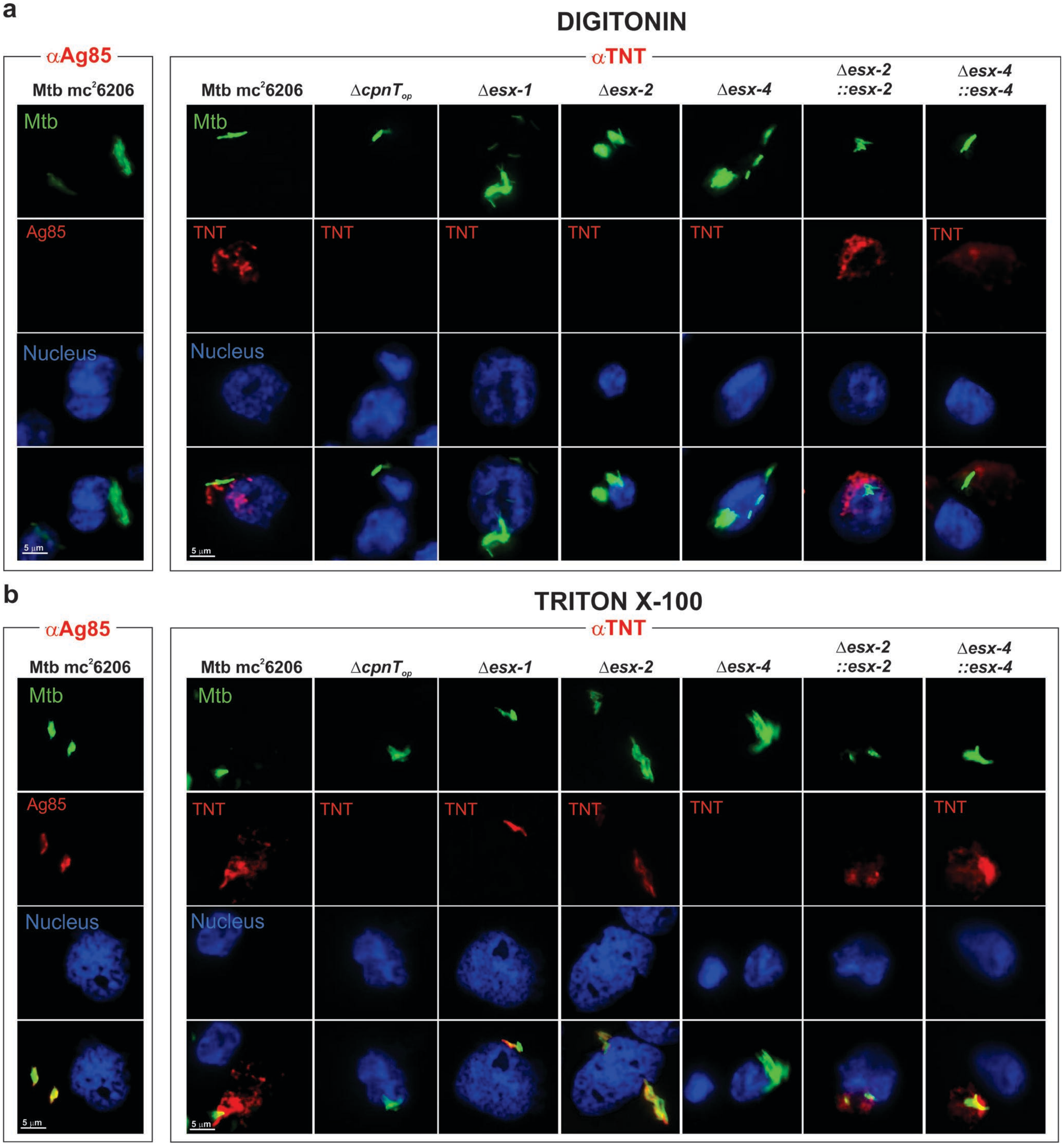

Supplementary Figure 14. The ESX-1, ESX-2 and ESX-4 systems are required for TNT translocation to the cytosol of macrophages infected with *M. tuberculosis*.
Full data set for Figure 4a with each fluorescence channel shown separately. Secretion of TNT into the cytosol of Mtb-infected macrophages. The indicated Mtb strains were labelled with DMN-Trehalose (green) and used to infect THP-1 macrophages at a MOI of 10:1. 48 h after infection, the macrophages were permeabilized with digitonin (**a**) to enable access of antibodies to the cytoplasm, or with Triton X-100 (**b**) for access to intracellular compartments. Then, cells were stained with an anti-TNT or anti-Ag85 antibody and with an Alexa Fluor-594 secondary antibody (red). The macrophage nuclei were stained with DAPI. The Δ*esx-1* strain has a deletion of the RD1 region and lacks the *panCD* genes. Shown are representative images from at least two independent experiments (n≥2).

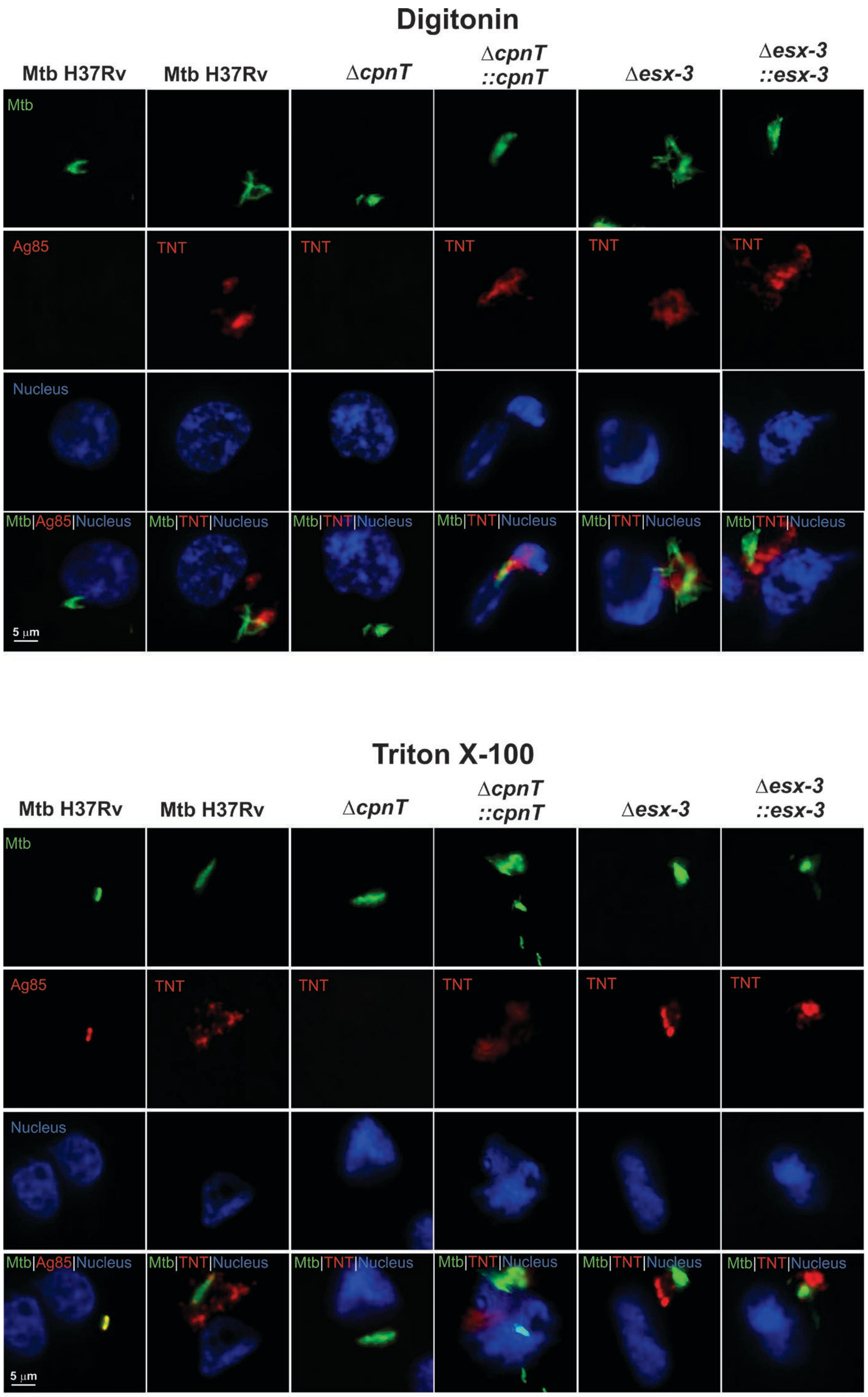

Supplementary Figure 15. The ESX-3 system is not required for the translocation of CpnT to the cytosol of macrophages infected with *M. tuberculosis.*
Full data set for Figure 4e with each fluorescence channel shown separately. Secretion of TNT into the cytosol of Mtb-infected macrophages. The indicated Mtb strains were labelled with DMN-Trehalose (green) and used to infect THP-1 macrophages at a MOI of 10:1. 48 h after infection, the macrophages were permeabilized with digitonin to enable access of antibodies to the cytoplasm, or with Triton X-100 for access to intracellular compartments. Then, cells were stained with an anti-TNT or anti-Ag85 antibody and with an Alexa Fluor-594 secondary antibody (red). The macrophage nuclei were stained with DAPI. Shown are representative images from at least two independent experiments (n≥2).

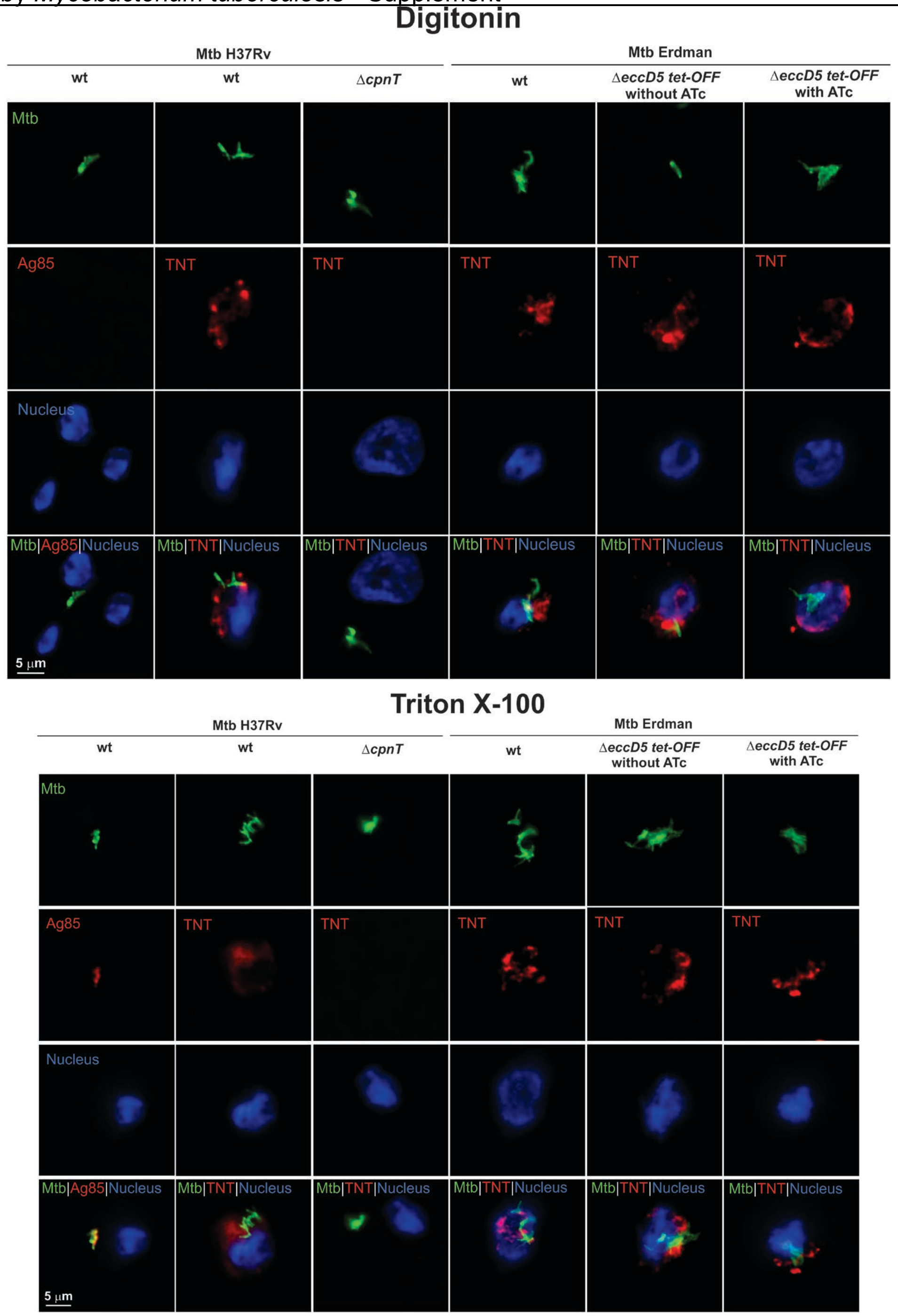

Supplementary Figure 16. The ESX-5 system is not required for the translocation of CpnT to the cytosol of macrophages infected with *M. tuberculosis.*

Full data set for Figure 4f with each fluorescence channel shown separately. Secretion of TNT into the cytosol of infected macrophages in the Mtb H37Rv and Erdman backgrounds. When indicated, bacteria were grown in 7H9 + 100 ng/ml anhydrotetracycline (ATc) to pre-deplete EccD5 prior to infection, then ATc was kept in the culture media during the infection. The indicated Mtb strains were labelled with DMN-Trehalose (green) and used to infect THP-1 macrophages at a MOI of 10:1. 48 h after infection, the macrophages were permeabilized with digitonin to enable access of antibodies to the cytoplasm, or with Triton X-100 for access to intracellular compartments. Then, cells were stained with an anti-TNT or anti-Ag85 antibody and with an Alexa Fluor-594 secondary antibody (red). The macrophage nuclei were stained with DAPI. Shown are representative images from at least two independent experiments (n≥2).

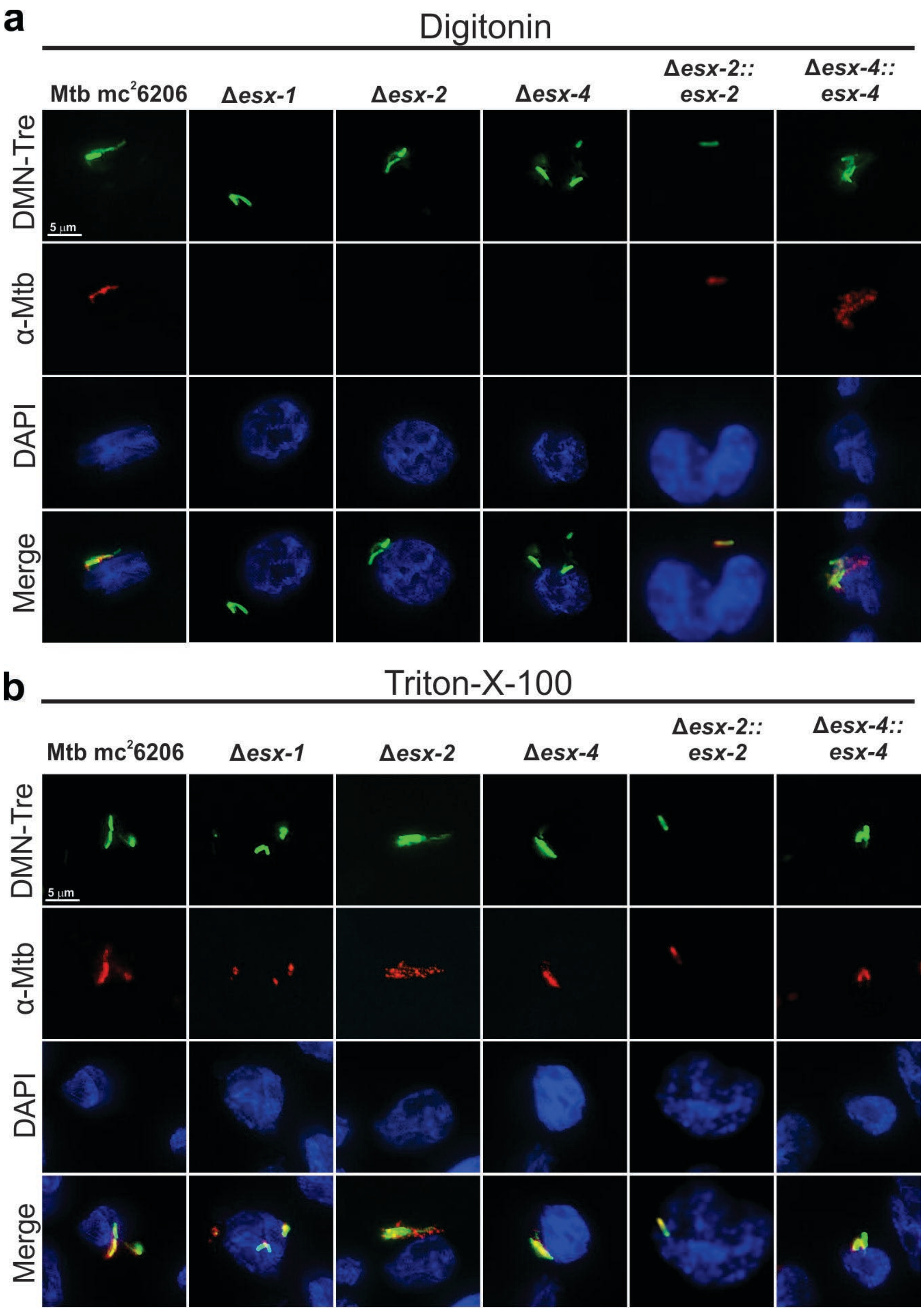

Supplementary Figure 17. The ESX-1, ESX-2 and ESX-4 systems are required for cytosolic access of *M. tuberculosis*.
Full data set for Figure 5a with each fluorescence channel shown separately. Immunofluorescent images of THP-1 macrophages infected with DMN-Tre labeled Mtb strains (green) for 48 hours and then stained with an anti-Mtb antibody and Alexa Fluor-594 secondary antibody (red). The macrophages were permeabilized with digitonin (**a**) to enable access of antibodies to the cytoplasm or with Triton X-100 (**b**) for access to intracellular compartments. The macrophage nuclei were stained with DAPI. The Δ*esx-1* strain has a deletion of the RD1 region and lacks the *panCD* genes. Shown are representative images from at least two independent experiments (n≥2).

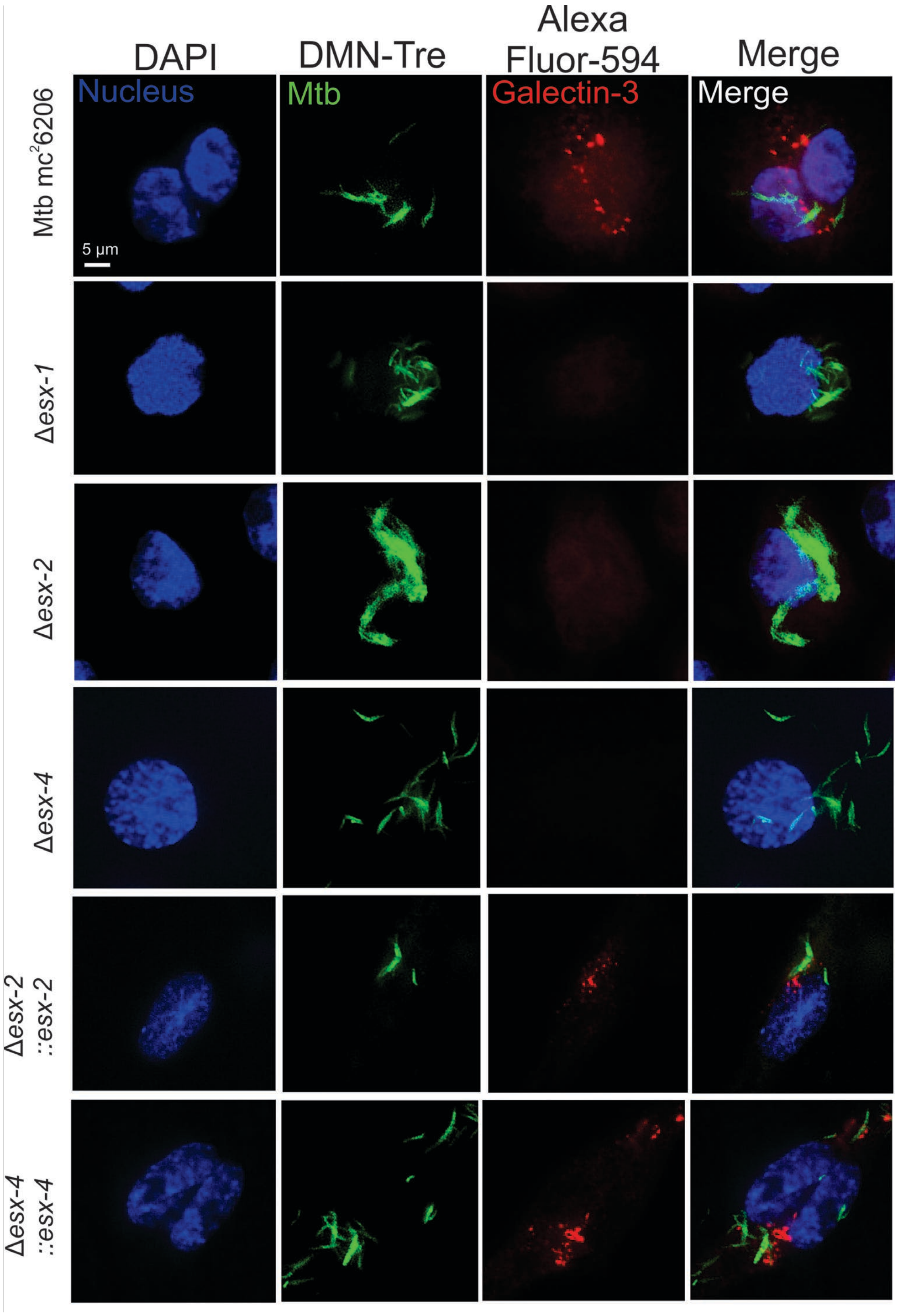

Supplementary Figure 18. The ESX-1, ESX-2 and ESX-4 systems are required for rupture of the phagosome by *M. tuberculosis*.

Full data set for Figure 5d with each fluorescence channel shown separately. Immunofluorescent images of THP-1 macrophages infected with DMN-Tre labeled Mtb strains (green) for 48 hours and then stained with an anti-Galectin-3 antibody and Alexa Fluor-594 secondary antibody (red). The macrophages were treated with Triton X-100 to permeabilize all intracellular compartments. The macrophage nuclei were stained with DAPI. The Δ*esx-1* strain has a deletion of the RD1 region and lacks the *panCD* genes. Shown are representative images from at least two independent experiments (n≥2).

Supplementary Tables

Strain name	Relevant genotypes and description	Source or Reference
M. tuberculosis mc^{2}6206	H37Rv derivative; Δ*leuCD* Δ*panCD*; avirulent *M. tuberculosis* (BSL-2)	[1]
M. tuberculosis ML2016	mc^{2}6206 derivative: Δ*esxF-esxE-cpnT-ift* (referred to as Δ*cpnT* operon)	[2]
M. tuberculosis ML2648	ML2016::pML3964	This study
M. tuberculosis ML2650	ML2016::pML3966	This study
M. tuberculosis ML2651	ML2016::pML3967	This study
M. tuberculosis ML2652	ML2016::pML3968	This study
M. tuberculosis ML2768	mc^{2}6206 derivative; Δ*eccC2* double crossover strain, marked	This study
M. tuberculosis ML2769	mc^{2}6206 derivative; Δ*eccC4* double crossover strain, marked	This study
M. tuberculosis ML2691	ML2768 derivative; Δ*eccC2;* unmarked	This study
M. tuberculosis ML2690	ML2769 derivative; Δ*eccC4*, unmarked	This study
M. tuberculosis ML2692	ML2691/cosmid I106 (contains *esx-2* operon)	This study
M. tuberculosis ML2696	ML2690/cosmid I60 (contains *esx-4* operon)	This study
M. tuberculosis mc^{2}6230	ΔRD1 Δ*panCD*; *esx-1* deletion mutant	[3]
M. tuberculosis H37Rv	wild-type	ATCC 25618
M. tuberculosis ML845	H37Rv derivative; Δ*cpnT,* unmarked	[4]
M. tuberculosis ML2001	ML845::pML3009	[4]
M. tuberculosis mc^{2}7788	H37Rv derivative; *esx-3* operon deletion mutant	[5]
M. tuberculosis mc^{2}7827	mc^{2}7788 derivative; *esx-3* operon deletion mutant complemented with *esx-3* cosmid at attB site	[5]
M. tuberculosis Erdman	wild-type	ATCC® 35801™
M. tuberculosis $eccD_5$ Tet-OFF	Mtb Erdman derivative; $eccD_5$ conditional mutant	[6]
E. coli MachI	*lac*ZΔM15, *hsd*R, *lac*X74, *rec*A, *end*A, *ton*A	Invitrogen

Supplementary Table 1. Strains used in this study

Plasmid	Properties	Reference
pML3009	PBR322 origin, *aph*, L5 attP, FRT-int-Pimyc-*esxF-esxEcpnT-rv3902c*-FRT	[4]
pML3042	*aph*, int_{L5}, $attP_{L5}$, psmyc-*esxF-esxE-*$cpnT_{HA}$*-ift;* $kan^{R;}$ integrative expression vector for the HA-marked *cpnT* operon	This study
pML3964	pML3042-derivative: $attP_{L5}$, psmyc-*esxF-esxE-cpnT-ift;* kan^{R} , integrative expression vector for the HA-marked *cpnT* operon	This study
pML3966	pML3964-derivative: $attP_{L5}$, psmyc-*esxF-esxE-*$cpnT_{Y88A}$*-ift;* kan^{R} , integrative *cpnT*-operon expression vector with mutation of ESX motif 1 (Y1*)	This study
pML3967	pML3964-derivative: $attP_{L5}$, psmyc-*esxF-esxE-*$cpnT_{Y741A}$*-ift;* kan^{R} , integrative *cpnT*-operon expression vector with mutation of ESX motif 2 (Y2*)	This study
pML3968	pML3964-derivative: $attP_{L5}$, psmyc-*esxF-esxE-cpnT-*$_{Y756A}$*-ift;* kan^{R}, integrative *cpnT*-operon expression vector with mutation of ESX motif 3 (Y3*)	This study
pML2424	pUC origin, pAL5000ts, *loxP*-psmyc-gfp_m^{2+}*-hyg-loxP*, pwmyc-*sacR-sacB;* hyg^{R} , vector for gene deletion in mycobacteria via homologous recombination	[7]
pML4245	pML2424-derivative; hom_{up}-*eccC2*- hom_{down}*;* hyg^{R}, *eccC2* deletion vector	This study
pML4255	pML2424-derivative; hom_{up}-*eccC4*- hom_{down}*;* hyg^{R}, *eccC4* deletion vector	This study
pML2714	pUC origin; pAL5000ts; P_{hsp60}*::cre*; P_{imyc}::*pamcherry1m*; *aph*; 7613 bp	[8]
Cosmid I106	pYUB412 cosmid, *bla*, *hyg,* int_{L5}, $attB_{L5}$; hyg^{R}.<br>coordinates of the integrated H37Rv DNA: ~4349-4385 kb<br>contains the ***esx-2* locus** of *M. tuberculosis* H37Rv	[9]
Cosmid I60	pYUB412 cosmid, *bla*, *hyg,* int_{L5}, $attB_{L5}$; hyg^{R}.<br>coordinates of the integrated H37Rv DNA: ~3848-3874 kb<br>contains the ***esx-4* locus** of *M. tuberculosis* H37Rv	[9]

Supplementary Table 2. Plasmids and cosmids used in this study

“Origin” means origin of replication. pAL5000ts denotes the temperature-sensitive origin of replication[10] of the pAL5000 plasmid[11]. The *bla*, *hyg* and *aph* genes confer resistance to ampicillin, hygromycin and kanamycin, respectively. The $attP_{L5}$ site is required for site specific integration of plasmids into the chromosomal *attB* site by the mycobacteriophage L5 integrase gene, int_{L5}. The site-specific recombinase Cre excises DNA fragments flanked by *loxP* sites. The constitutive mycobacterial promoters p_{smyc} and p_{imyc} have been described previously[12,13]. We cloned app. 1000 bp of homologous up- and downstream sequences to flank the *loxP*-psmyc-gfp_m^{2+}-*hyg*-*loxP* cassette of pML2424 for the construction of the *eccC2* and *eccC4* deletion vectors pML4245 and pML4255. Upstream and downstream sequences were cloned using SpeI/SwaI and PacI/NsiI, respectively.

Oligonucleotide	Sequence
EsxF-PacI-FWD	ATATTTAATTAACAGAAAGGAGGATTTCAACTATCATGGGTGCCGACGACACGC
IFT-Rev-HindIII	GCAAGCTTTAACCCTTATAGTCCTTCCAAA
CpnT M1 Y88A Fwd	AGCGCGCACAACGCCTCGTTG
CpnT M1 Y88A Rev	CAACGAGGCGTTGTGCGCGCT
CpnT M2 Y356A Fwd	TTCGGGTGGGCCCGCTCCTGG
CpnT M2 Y356A Rev	CCAGGAGCGGGCCCACCCGAA
CpnT M3 Y741A Fwd	AGGATCGCCGCCACTAATCTC
CpnT M3 Y741A Rev	GAGATTAGTGGCGGCGATCCT
eccC2-DN-Nsi-r	ATTATATATGCATCGAGAACCCCTCCGTAGGTG
eccC2-inner-f	AGGCGCAGCTCACGCTGATCGAC
EccC2-UP-SPE-f	ATATAGACTAGTGAGATCGCCAAGTATCCGAC
EccC2-UP-Swa-r	CTCTCAATTTAAATGGAAACGCTTTCTTGGACAT
EccC4-DN-nsiI-Rev	AGAGATATGCATGTGATTGTGTGACGTGCAAC
EccC4-HindIII-Rev	GCAGAATTCGAAGCTTCTACTGCTCGCCGGGCACCGACGG
EccC4-inn-f	TCGACGTCATCGAGTCGGAACA
EccC4-Inner-Fwd	GCACACGCGACCATTGCGGATGCGCCCATCGCGATCCCCTTGCGGGTCGG
EccC4-Inner-Rev	ACATGTACGCCGAATGAAAGCCCTTGAGCTGCCAGGGCAACGATCGACTCCTCAAGGGC
EccC4-UP-Spe-F	AGAGATACTAGTCGGATACCTCTAACAGACCA
EccC4-UP-Swa-r	CTCTCAATTTAAATGCTTGTGGACAACCGAATAC

Supplementary Table 3. Oligonucleotides used in this study

**Antibody**	**Source**
Rabbit polyclonal anti-TNT	Generated by external vendor[4]
Rabbit polyclonal anti-IFT	Generated by external vendor[14]
Rabbit polyclonal anti-EsxF	Generated by external vendor[2]
Rabbit polyclonal anti-*Mycobacterium tuberculosis*	Commercial - Ab905, Abcam:
Mouse monoclonal anti-LpqH (clone IT-54)	Commercial - NR-13792; BEI Resources
Rabbit polyclonal anti-Antigen-85 complex	Commercial - NR-13800; BEI Resources
Rabbit polyclonal anti-CFP-10	Commercial - NR-13801; BEI Resources
Mouse monoclonal anti-Mctb (clone 5D1.23)	Generated by external vendor[15]
Rabbit polyclonal anti-Glpx	Generated by external vendor[2]
Rabbit polyclonal anti-PPE41	Generated by external vendor[6]
Rabbit polyclonal anti-$EccD_5$	Generated by external vendor[6]
Mouse monoclonal anti-Galectin-3 (clone A3A12)	Commercial - Ab2785, Abcam
Donkey anti-rabbit IgG (H+L)-Alexa Fluor-594 secondary	Commercial - A-21207, Thermo Fisher
Donkey anti-rabbit IgG (H+L)-Alexa Fluor-488	Commercial - A-21206, Thermo Fisher
IRDye 680RD Donkey anti-rabbit IgG secondary	Commercial - 926-68073, LI-COR
IRDye 800CW Goat anti-mouse IgG Secondary	Commercial - 926-332210, LI-COR

Supplementary Table 4. Antibodies used in this study

Uncropped scans of blots shown in figures

Supplementary Figure 3

Immunoblots:
Subcellular fractionation of Mtb mc[2]6206 and derivative strains, showing the membrane and soluble fractions.

1. mc[2]6206 Δ*esxFE-cpnT-ift* + *esxFE-cpnT-ift* (membrane fraction)
2. mc[2]6206 Δ*esxFE-cpnT-ift* + *esxFE-cpnT-ift* (soluble fraction)
3. mc[2]6206 Δ*esxFE-cpnT-ift* (membrane fraction)
4. mc[2]6206 Δ*esxFE-cpnT-ift* (soluble fraction)
5. mc[2]6206 Δ*esxFE-cpnT-ift* + *esxFE-cpnT-Y1*-ift* (membrane fraction)
6. mc[2]6206 Δ*esxFE-cpnT-ift* + *esxFE-cpnT-Y1*-ift* (soluble fraction)
7. mc[2]6206 Δ*esxFE-cpnT-ift* + *esxFE-cpnT-Y2*-ift* (membrane fraction)
8. mc[2]6206 Δ*esxFE-cpnT-ift* + *esxFE-cpnT-Y2*-ift* (soluble fraction)
9. mc[2]6206 Δ*esxFE-cpnT-ift* + *esxFE-cpnT-Y3*-ift* (membrane fraction)
10. mc[2]6206 Δ*esxFE-cpnT-ift* + *esxFE-cpnT-Y3*-ift* (soluble fraction)

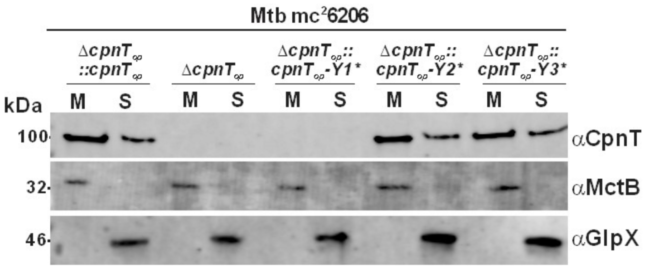

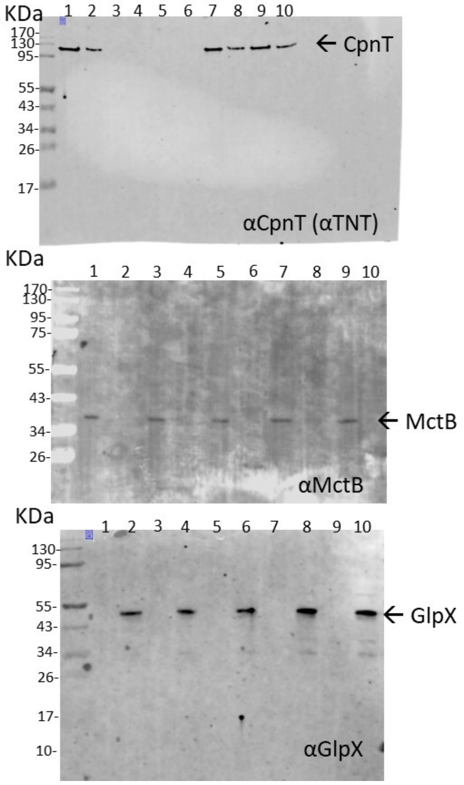

Supplementary Figure 8

Immunoblots:
Whole cell lysates (CL) and culture filtrates (CF) of Mtb mc[2]6206 and derivative strains

1. mc[2]6206 (whole cell lysate)
2. mc[2]6206 (culture filtrate)
3. mc[2]6206 Δ*esx-1* (whole cell lysate)
4. mc[2]6206 Δ*esx-1* (culture filtrate)
5. mc[2]6206 Δ*esx-2* (whole cell lysate)
6. mc[2]6206 Δ*esx-2* (culture filtrate)
7. mc[2]6206 Δ*esx-4* (whole cell lysate)
8. mc[2]6206 Δ*esx-4* (culture filtrate)
9. mc[2]6206 Δ*esx-2::esx-2* (whole cell lysate)
10. mc[2]6206 Δ*esx-2::esx-2* (culture filtrate)
11. mc[2]6206 Δ*esx-4::esx-4* (whole cell lysate)
12. mc[2]6206 Δ*esx-4::esx-4* (culture filtrate)

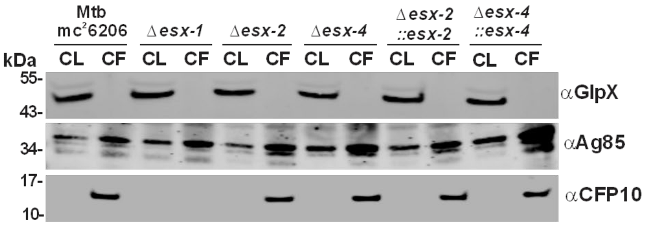

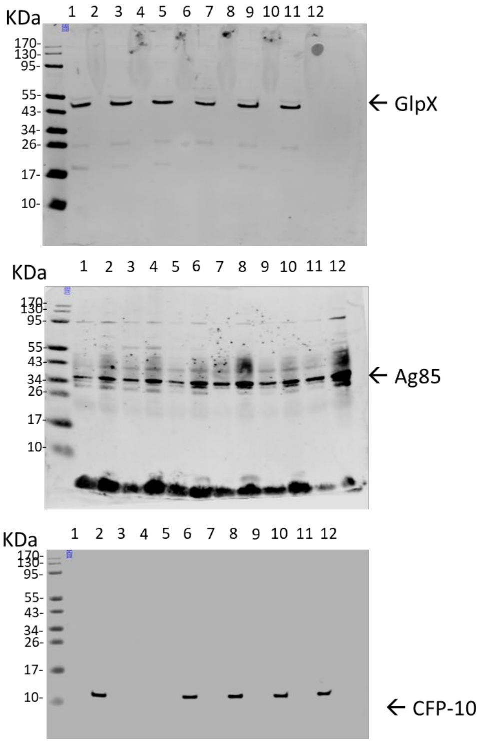

Supplementary Figure 10

Immunoblots:
Subcellular fractionation of Mtb mc[2]6206 and derivative strains, showing the membrane and soluble fractions.

1. mc[2]6206 (membrane fraction)
2. mc[2]6206 (soluble fraction)
3. mc[2]6206 Δ*esxFE-cpnT-ift* (membrane fraction)
4. mc[2]6206 Δ*esxFE-cpnT-ift* (soluble fraction)
5. mc[2]6206 Δ*esx-1* (membrane fraction)
6. mc[2]6206 Δ*esx-1* (soluble fraction)
7. mc[2]6206 Δ*esx-2* (membrane fraction)
8. mc[2]6206 Δ*esx-2* (soluble fraction)
9. mc[2]6206 Δ*esxG* (membrane fraction)
10. mc[2]6206 Δ*esxG* (soluble fraction)
11. mc[2]6206 Δ*esx-4* (membrane fraction)
12. mc[2]6206 Δ*esx-4* (soluble fraction)
13. mc[2]6206 Δ*esx-4::esx-4* (membrane fraction)
14. mc[2]6206 Δ*esx-4::esx-4* (soluble fraction)

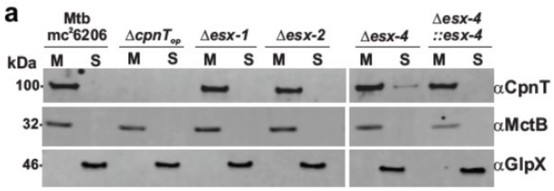

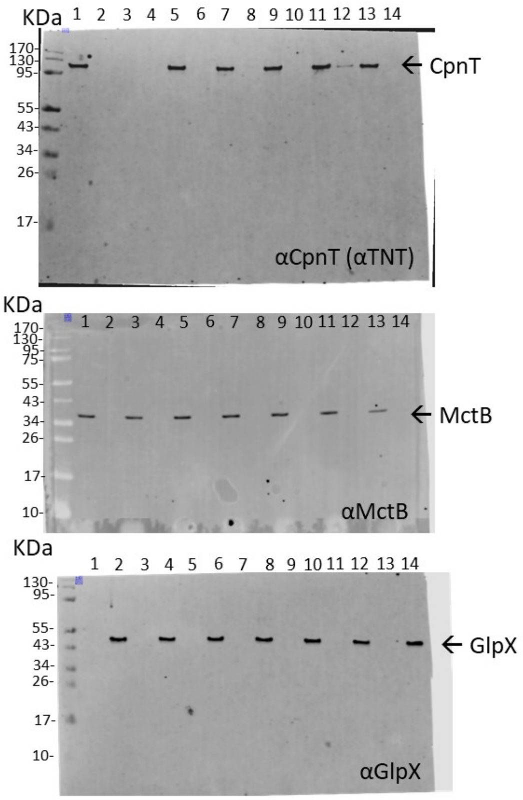

Supplementary References

1 Sampson, S. L. *et al.* Protection elicited by a double leucine and pantothenate auxotroph of *Mycobacterium tuberculosis* in guinea pigs. *Infect Immun* **72**, 3031-3037, (2004).

2 Tak, U., Dokland, T. & Niederweis, M. Pore-forming Esx proteins mediate toxin secretion by *Mycobacterium tuberculosis*. *Nat Commun* **12**, 394, (2021).

3 Sambandamurthy, V. K. *et al. Mycobacterium tuberculosis ∆RD1 ∆panCD*: a safe and limited replicating mutant strain that protects immunocompetent and immunocompromised mice against experimental tuberculosis. *Vaccine* **24**, 6309-6320, (2006).

4 Danilchanka, O. *et al.* An outer membrane channel protein of *Mycobacterium tuberculosis* with exotoxin activity. *Proc Natl Acad Sci U S A* **111**, 6750-6755, (2014).

5 Tufariello, J. M. *et al.* Separable roles for *Mycobacterium tuberculosis* ESX-3 effectors in iron acquisition and virulence. *Proc Natl Acad Sci U S A* **113**, E348-357, (2016).

6 White, D. W., Elliott, S. R., Odean, E., Bemis, L. T. & Tischler, A. D. *Mycobacterium tuberculosis* Pst/SenX3-RegX3 Regulates Membrane Vesicle Production Independently of ESX-5 Activity. *MBio* **9**, e00778, (2018).

7 Mitra, A., Speer, A., Lin, K., Ehrt, S. & Niederweis, M. PPE surface proteins are required for heme utilization by *Mycobacterium tuberculosis*. *MBio* **8**, e01720, (2017).

8 Ofer, N. *et al.* Ectoine biosynthesis in *Mycobacterium smegmatis*. *Appl Environ Microbiol* **78**, 7483-7486, (2012).

9 Philipp, W. J. *et al.* An integrated map of the genome of the tubercle bacillus, *Mycobacterium tuberculosis* H37Rv, and comparison with *Mycobacterium leprae*. *Proc Natl Acad Sci U S A* **93**, 3132-3137, (1996).

10 Guilhot, C., Gicquel, B. & Martin, C. Temperature-sensitive mutants of the *Mycobacterium* plasmid pAL5000. *FEMS Microbiol Lett* **77**, 181-186, (1992).

11 Labidi, A., David, H. L. & Roulland-Dussoix, D. Restriction endonuclease mapping and cloning of *Mycobacterium fortuitum var. fortuitum* plasmid pAL5000. *Ann Inst Pasteur Microbiol* **136B**, 209-215, (1985).

12 Kaps, I. *et al.* Energy transfer between fluorescent proteins using a co-expression system in *Mycobacterium smegmatis*. *Gene* **278**, 115-124, (2001).

13 Mailaender, C. *et al.* The MspA porin promotes growth and increases antibiotic susceptibility of both *Mycobacterium bovis* BCG and *Mycobacterium tuberculosis*. *Microbiology* **150**, 853-864, (2004).

14 Sun, J. *et al.* The tuberculosis necrotizing toxin kills macrophages by hydrolyzing NAD. *Nat Struct Mol Biol* **22**, 672-678, (2015).

15 Wolschendorf, F. *et al.* Copper resistance is essential for virulence of *Mycobacterium tuberculosis*. *Proc Natl Acad Sci U S A* **108**, 1621-1626, (2011).
